# Exploring GZMK as a prognostic marker and predictor of immunotherapy response in breast cancer: unveiling novel insights into treatment outcomes

**DOI:** 10.1007/s00432-024-05791-6

**Published:** 2024-06-04

**Authors:** Zitao Li, Qiqi Xie, Fuxing Zhao, Xinfa Huo, Dengfeng Ren, Zhilin Liu, Xiaofeng Zhou, Guoshuang Shen, Jiuda Zhao

**Affiliations:** 1 Research Center for High Altitude Medicine, Key Laboratory of High Altitude Medicine (Ministry of Education), Key Laboratory of Application and Foundation for High Altitude Medicine Research in Qinghai Province (Qinghai-Utah Joint Research Key Lab for High Altitude Medicine), Laboratory for High Altitude Medicine of Qinghai Province, Qinghai University, Xining, 810000 China; 2 Breast Disease Diagnosis and Treatment Center of Qinghai University Affiliated Hospital & Affiliated Cancer Hospital of Qinghai University, Xining, 810000 China; 3 Pathology Department, Affiliated Hospital of Qinghai University, Xining, 810000 China

**Keywords:** GZMK, Prognosis, Immunotherapy, Breast cancer, Apoptosis

## Abstract

**Background:**

Granzyme K (GZMK) is a crucial mediator released by immune cells to eliminate tumor cells, playing significant roles in inflammation and tumorigenesis. Despite its importance, the specific role of GZMK in breast cancer and its mechanisms are not well understood.

**Methods:**

We utilized data from the TCGA and GEO databases and employed a range of analytical methods including GO, KEGG, GSEA, ssGSEA, and PPI to investigate the impact of GZMK on breast cancer. In vitro studies, including RT-qPCR, CCK-8 assay, cell cycle experiments, apoptosis assays, Celigo scratch assays, Transwell assays, and immunohistochemical methods, were conducted to validate the effects of GZMK on breast cancer cells. Additionally, Cox regression analysis integrating TCGA and our clinical data was used to develop an overall survival (OS) prediction model.

**Results:**

Analysis of clinical pathological features revealed significant correlations between GZMK expression and lymph node staging, differentiation grade, and molecular breast cancer subtypes. High GZMK expression was associated with improved OS, progression-free survival (PFS), and recurrence-free survival (RFS), as confirmed by multifactorial Cox regression analysis. Functional and pathway enrichment analyses of genes positively correlated with GZMK highlighted involvement in lymphocyte differentiation, T cell differentiation, and T cell receptor signaling pathways. A robust association between GZMK expression and T cell presence was noted in the breast cancer tumor microenvironment (TME), with strong correlations with ESTIMATEScore (Cor = 0.743, *P* < 0.001), ImmuneScore (Cor = 0.802, *P* < 0.001), and StromalScore (Cor = 0.516, *P* < 0.001). GZMK also showed significant correlations with immune checkpoint molecules, including CTLA4 (Cor = 0.856, *P* < 0.001), PD-1 (Cor = 0.82, *P* < 0.001), PD-L1 (Cor = 0.56, *P* < 0.001), CD48 (Cor = 0.75, *P* < 0.001), and CCR7 (Cor = 0.856, *P* < 0.001). Studies indicated that high GZMK expression enhances patient responsiveness to immunotherapy, with higher levels observed in responsive patients compared to non-responsive ones. In vitro experiments confirmed that GZMK promotes cell proliferation, cell division, apoptosis, cell migration, and invasiveness (*P* < 0.05).

**Conclusion:**

Our study provides insights into the differential expression of GZMK in breast cancer and its potential mechanisms in breast cancer pathogenesis. Elevated GZMK expression is associated with improved OS and RFS, suggesting its potential as a prognostic marker for breast cancer survival and as a predictor of the efficacy of immunotherapy.

**Supplementary Information:**

The online version contains supplementary material available at 10.1007/s00432-024-05791-6.

## Introduction

Recent statistics forecast that breast cancer will affect 313,510 individuals in the United States in 2024, with an estimated 42,780 fatalities. Breast cancer remains the most prevalent malignancy among women, accounting for approximately 32% of all cancer cases (Siegel et al. 2024). Despite advances in early screening and comprehensive treatments—including surgery, radiotherapy, chemotherapy, endocrine therapy, and targeted therapy, which have reduced recurrence and metastasis rates—nearly all patients with metastatic breast cancer eventually succumb to the disease (Nolan et al. 2023). This underscores the urgent need for novel therapeutic approaches to reduce the mortality and metastasis rates of breast cancer.

Immunotherapy, including therapies such as chimeric antigen receptor T cell therapy (CAR-T), cancer vaccines, oncolytic viruses, and immune checkpoint inhibitors (ICB), is transforming the treatment landscape of various solid tumors (Meric-Bernstam et al. 2021; Agache et al. 2022). Among these, ICB agents like inhibitors of cytotoxic T lymphocyte-associated antigen-4 (CTLA-4), programmed cell death protein-1 (PD-1), and programmed cell death-ligand 1 (PD-L1) have gained widespread clinical application (Yap et al. 2021). Notably, PD-L1 inhibitors have shown promising efficacy in triple-negative breast cancer (TNBC), as evidenced by the Impassion 130 study which reported a median overall survival of 25 months in PD-L1-positive locally advanced or metastatic TNBC patients treated with atezolizumab in combination with nab-paclitaxel, resulting in a 7-month improvement in overall survival, with manageable side effects (Emens et al. 2021).

Most immunotherapies aim to enhance the cytotoxicity of lymphocytes, primarily focusing on cytotoxic T cells (Tc) and natural killer cells (NK), to effectively kill cancer cells and eradicate tumors (Yap et al. 2021). Tc cells are pivotal in this process, inducing tumor cell death through two main pathways: the perforin/granzyme pathway and the death ligand/receptor pathway (Lowin et al. 1994). Among these, the perforin/granzyme pathway is both rapid and predominant, where perforin facilitates the entry of granzymes into target cells, which then swiftly cleave various substrates in the cytoplasm and nucleus, including nuclear assembly protein SET, hnRNP K, P53, β-tubulin, and α-tubulin, thereby inducing cell death (Hua et al. 2009; Domselaar and Quadir 2012; Zhao et al. 2007a). Granzymes are serine proteases primarily involved in promoting the apoptosis of infected or tumor cells. Humans possess five granzymes: granzyme A (GZMA), GZMB, GZMH, GZMK, and GZMM (Bots and Medema 2006). Although the apoptosis mechanisms mediated by GZMA and GZMB are well-documented, the roles of other granzymes, especially GZMK, which exhibits trypsin-like catalytic activity, are less understood in Tc-mediated cytotoxicity, particularly in breast cancer (Camell 2020). Therefore, further research into the molecular mechanisms of granzyme-induced apoptosis, especially the role of GZMK in breast cancer, is crucial for advancing our understanding of breast cancer immunotherapy and enhancing the reliability of clinical evidence for its treatment.

Despite the promising efficacy of immunotherapy, only a minority of patients benefit from it (Cai et al. 2022). For example, the response rate among melanoma patients, who are among the best responders to immunotherapy, is approximately 20%, which can increase to about 30–50% with combination therapy (Meric-Bernstam et al. 2021). Consequently, effective screening for immune-related biomarkers associated with treatment efficacy is imperative. Although potential biomarkers such as PD-L1 expression levels, tumor mutation burden (TMB), and tumor-infiltrating T cells have been identified, their clinical validation remains contentious (Yap et al. 2021; Khan et al. 2023; Jardim et al. 2021). Moreover, these biomarkers often prove difficult to translate into clinical practice, further limiting their practical application. Hence, a deeper exploration of their feasibility and efficacy in clinical settings is warranted.

## Materials and methods

### Data acquisition and processing

The Cancer Genome Atlas (TCGA, https://cancergenome.nih.gov/) provides publicly available cancer genomic datasets. Using R language, we downloaded the TPM (Transcripts Per Million) data of breast cancer (BRCA) transcriptomes from the TCGA database (https://portal.gdc.cancer.gov). This dataset includes 112 samples of adjacent normal breast tissue and 1085 samples of breast cancer tissue. Additionally, we obtained the follow-up and clinical data corresponding to the TCGA breast cancer from the UCSC Xena website. For genes with duplicate names, we averaged their values. Genes with an average expression level below 0.05 were excluded, and the expression levels of the remaining genes were retained for analysis. The expression levels of genes in each sample were log-transformed (log2(x + 1)) and then standardized. Breast cancer datasets GSE11121, GSE12093, GSE162228, GSE17705, GSE20685, GSE20711, GSE21653, GSE22219, GSE25055, GSE25065, GSE42568, GSE45255, GSE48390, GSE58812, GSE61304, GSE7390, GSE88770, GSE97342, and GSE9893 were all obtained from the Gene Expression Omnibus (https://www.ncbi.nlm.nih.gov/geo/).

### Patient source and clinical pathological features

We obtained clinical data of breast cancer patients from the TCGA BRCA project and the Pathology Department of Qinghai University Affiliated Hospital. The Ethics Committee of Qinghai University Affiliated Hospital approved this study, Approval No.: P-SL-202242. Patients were informed and gave their consent to participate in this study. We collected data from 131 BRCA patients diagnosed at Qinghai University Affiliated Hospital between 2020 and 2023, along with 6 normal control paraffin-embedded specimens. No treatment was administered before surgery. We statistically analyzed the clinical pathological characteristics of these patients and examined their correlation with GZMK expression using the R package gtsummary [version 1.7.2]. A multivariable Cox regression analysis was conducted to assess the impact of clinical pathological features and GZMK expression on the overall survival (OS) of breast cancer patients. We constructed prognostic prediction models for 3-year, 5-year, and 8-year OS using the rms package [version 6.2–0] and survival package [version 3.2–10], and displayed these models as nomograms. Additionally, we plotted calibration curves using the rms package [version 6.20] and survival package [version 3.2–10] to evaluate the accuracy of the prediction model.

### Differential gene and prognosis-related gene selection

The original TCGA data were analyzed using the limma package after normalization. Differentially expressed genes (DEGs) were identified using the criteria of |log2 fold change (log2FC)|> 1 and an adjusted *P*-value < 0.05. Single-factor Cox regression was employed to identify genes related to the prognosis of breast cancer. The VennDiagram package was then used to intersect the DEGs and prognosis-related genes, and the results were visualized with a Venn diagram.

### Functional enrichment and pathway analysis

Gene Ontology (GO) and Kyoto Encyclopedia of Genes and Genomes (KEGG) pathway enrichment analyses were conducted on intersecting genes using the clusterProfiler package (Wu et al. 2021a) (version 4.10.1). Gene Set Enrichment Analysis (GSEA) was performed. The GSVA package (version 1.34.0) was used to score the pathway activities between groups with high and low GZMK expression (divided by the mean). T-tests calculated the differences in pathway activities between these groups, with a cutoff of *P* < 0.05.

### Protein–protein interaction network construction and key gene selection

The Search Tool for the Retrieval of Interacting Genes (STRING) (http://string-db.org; version: 11.0) predicted protein–protein interaction (PPI) networks online. The network was visualized using Cytoscape software, and hub genes were identified with the cytoHubba plugin. Initially, the intersection DEGs were analyzed in STRING and subsequently visualized in a PPI network using Cytoscape.

### GZMK survival analysis

Kaplan–Meier survival curves were analyzed for significance using the Log-rank test, with *P* < 0.05 indicating statistical significance. The Kaplan Meier database (http://kmplot.com/) was used to assess the impact of GZMK on the prognosis of breast cancer and its subgroups.

### Immunohistochemical analysis of GZMK expression levels in breast cancer and normal tissues

The immunohistochemistry (IHC) protocol for GZMK expression analysis involved baking tissue cores at 60 °C for 30 min, deparaffinization, antigen retrieval using the citrate method, peroxidase blocking, incubation with primary and secondary antibodies, and staining with DAB solution. Slides were counterstained, dehydrated, and mounted. After air-drying, the slides were scanned for result observation. The original method for data interpretation assessed staining intensity (0/1 + /2 + /3 +) and positivity rate for cytoplasmic and membranous staining separately for each sample group. Standardization involved scoring staining intensity as 0 (negative), 1 (I), 2 (II), 3 (III); and staining positivity as 0 (negative), 1 (1–25%), 2 (26–50%), 3 (51–75%), 4 (76–100%). GZMK total scores were calculated by multiplying the staining intensity score by the staining positivity score, and significant differences in expression levels among groups were analyzed using t-tests.

### Cell culture and transfection

The MCF-10A, SKBR3, MDA-MB-468, HCC1937, and MDA-MB-231 cell lines were obtained from the Cell Bank of the Chinese Academy of Sciences. All cell lines, except MCF-10A, were cultured in RPMI 1640 medium (Gibco) supplemented with 10% fetal bovine serum (FBS, Gibco; Thermo Fisher Scientific) and 1% penicillin/streptomycin solution (HyClone, Cytiva). MCF-10A cells were cultured in DMEM-F12 medium (Gibco) containing 10% FBS, 0.3 g/L L-glutamine, 20 ng/mL epidermal growth factor (EGF), 10 μg/mL insulin, 500 μg/mL hydrocortisone, and 40 mg/L gentamicin. All cells were maintained at 37 °C in a 5% CO2 atmosphere. The human GZMK cDNA was cloned into the GV492 lentiviral vector to construct the GZMK overexpression vector. Cells were transfected with the LV-GZMK (28,597–1) virus, and 72 h later, fluorescence microscopy confirmed that the infection efficiency was above 80%, suitable for subsequent experiments.

### Real-time quantitative PCR detection of GZMK expression levels post-transfection

Real-time quantitative PCR (RT-qPCR) was performed to assess GZMK expression levels in MDA-MB-231 cells following viral transfection. The Real-time Quantitative PCR Detecting System used ACTB as the internal reference gene. The upstream primer sequence for ACTB was GCGTGACATTAAGGAGAAGC, and the downstream primer sequence was CCACGTCACACTTCATGATGG, with an amplification fragment length of 236 bp. For GZMK, the upstream primer sequence was AACAGCCAAAGTTACTACAA, the downstream primer sequence was CCCTGAGTCACCCTTACAG, and the amplification fragment length was 102 bp. The relative quantification analysis was performed using the formula F = 2^−ΔΔCt^.

### CCK-8 assay

Cells from each experimental group, in the logarithmic growth phase, were trypsinized, resuspended in complete culture medium, and counted. The density for plating was established based on the growth rate, typically at about 2000 cells per well in a 100 μl volume. Each group was plated in triplicate to quintuplicate, according to the experimental design. For instance, if the experiment spanned five days, five plates, each with 96 wells, were prepared. After uniform preparation of all plates, cells were allowed to settle completely. Cell density in each experimental group was then observed under a microscope. If uneven density was noted, adjustments were made in one group and the cell quantities in the other groups were modified accordingly before re-plating. For example, if the control group exhibited a higher cell density, the number of cells was reduced before re-plating. The plates were then incubated in a cell culture incubator. Starting from the second day post-plating, 10 μl of CCK-8 reagent was added to each well 2–4 h before the incubation period ended, without changing the medium. After 2–4 h of incubation with CCK-8, the 96-well plates were shaken for 2–5 min, and the OD values were measured at 450 nm using a microplate reader. The data were then subjected to statistical analysis.

### Cell cycle analysis

For adherent cells, when confluence reached approximately 80%—before entering the plateau phase of growth—cells were trypsinized, resuspended in complete culture medium, and collected in 5 ml centrifuge tubes. Each group had three replicate wells prepared, ensuring an adequate cell count for analysis (at least 10^6^ cells per treatment). Suspension cells were collected directly. After collection, cells were centrifuged at 1300 rpm for 5 min, the supernatant was discarded, and the cell pellet was washed once with pre-chilled DPBS (pH = 7.2 ~ 7.4) at 4 °C. Following another centrifugation at the same speed and duration, a cell staining solution was prepared in the following ratio: 40 × PI stock solution (2 mg/ml): 100 × RNase stock solution (10 mg/ml): 1 × DPBS: 25 × Triton X-100 = 25: 10: 1000: 40. A suitable volume of this staining solution, between 0.6–1 ml depending on the cell quantity, was added and thoroughly mixed to achieve a cell flow rate of 300 ~ 800 cells/s during analysis. Cell cycle analysis was subsequently performed using flow cytometry, with data analysis facilitated by ModFit software.

### Apoptosis detection

When cell confluence in each experimental group reached about 70% in a 6-well plate, apoptosis was induced through drug treatment. For adherent cells, both supernatant and adherent cells were collected. Cells were trypsinized, resuspended in complete culture medium, and gathered in the same 5 ml centrifuge tube as the supernatant cells. Each group had three replicate wells, ensuring a sufficient cell count for analysis (at least 5 × 10^5^ cells per treatment). Suspension cells were collected directly. After collection, the cells were centrifuged at 1300 rpm for 5 min, the supernatant was discarded, and the cell pellet was washed once with pre-chilled PBS at 4 °C. Following a wash with 1 × binding buffer and a subsequent centrifugation at 1300 rpm for 3 min, the cells were collected. The cell pellet was then resuspended in 200 μl of 1 × binding buffer, to which 10 μl of Annexin V-APC staining solution was added. The cells were incubated at room temperature in the dark for 10–15 min. Depending on the cell quantity, an additional 400-800 μl of 1 × binding buffer was added. The cells were then analyzed by flow cytometry within 15 min in the dark. The results were analyzed accordingly.

### Celigo scratch assay

Cells from each experimental group, in the logarithmic growth phase, were trypsinized, resuspended in complete culture medium, and subsequently counted. The cell density for plating was established based on the size of the cells, targeting a density of 50,000 cells per well to achieve more than 90% confluence by the following day. The cells were cultured in a 37 °C incubator with 5% CO2, each group having three replicate wells with 100 μL of culture volume per well. The next day, a scratch was carefully made by gently pushing upwards with a scratch tool aligned with the central upper part of the 96-well plate. After creating the scratch, the plate was rinsed gently 2–3 times with PBS, and serum-containing medium (1% FBS) was added. The initial scan of the plate was performed at 0 h. The cells continued to be cultured under the same conditions, and suitable time points for scanning with Celigo were selected based on the degree of healing, with a total of three scans conducted. The migration area analysis using Celigo included several steps: (A) Celigo scanned and captured images of the targeted 96-well plate at 0 h and subsequent time points. (B) Celigo analyzed these images to measure the area covered by cells. (C) The migration rate was calculated using the formula [(S3 + S4)—(S1 + S2)] / [1—(S1 + S2)]. D) Cells with a migration rate greater than 30% at 24 h were considered to demonstrate good migration ability, deeming them suitable for downstream experiments.

### Transwell migration assay

Transwell inserts were removed from the kit and placed into a new 24-well plate as required. A volume of 100 µl of serum-free culture medium was added to the upper chamber of each insert, and the assembly was incubated at 37 °C for 1 h. A cell suspension was prepared in serum-free medium, cells are counted, and their number was adjusted based on previous experimental findings, typically to 10^5^ cells per well. The culture medium was then carefully removed from the upper chamber, and 100 µl of the cell suspension was added. Additionally, 600 µl of medium containing 30% FBS was added to the lower chamber. The setup was incubated at 37 °C for a duration determined by preliminary experimental results, typically allowing for a 16-h incubation before one insert was removed for staining and observation. The removal times of subsequent inserts were adjusted based on the observed degree of cell migration, thereby determining the total experimental duration. Each insert was then inverted onto absorbent paper to remove excess medium, and non-migratory cells were gently removed from the upper surface using a cotton swab. The inserts were fixed in 4% paraformaldehyde for 30 min, excess fixative was blotted off with absorbent paper, and 1–2 drops of staining solution were applied to the underside of the membrane. Migrated cells were stained for 1–3 min. After staining, the inserts were rinsed several times with PBS and allowed to air-dry. For microscopic imaging, fields of view were randomly selected for each Transwell insert, capturing four images at 100X magnification and nine images at 200X magnification. Cells in the 200X images were counted for data analysis. The number of migrated cells per field, the standard deviation, and a T-test were calculated to assess whether there is a significant difference in migratory ability between the experimental and control groups (*P* < 0.05 indicates statistical significance).

### Data analysis

Statistical analysis and visualization of all data from public databases were performed using R software (version 4.1.0). The significance of differences was determined using the Wilcoxon signed-rank test for two groups and the Kruskal–Wallis test for more than two groups. Pearson correlation was used to evaluate all correlations. Experiments were conducted in triplicate, and statistical significance was calculated using R software (version 4.1.0), with a significance level set at *P* < 0.05.

## Results

### Relative expression analysis of GZMK in breast cancer tissues and normal epithelial tissues and expression level analysis in cell lines

**Fig. 1:**
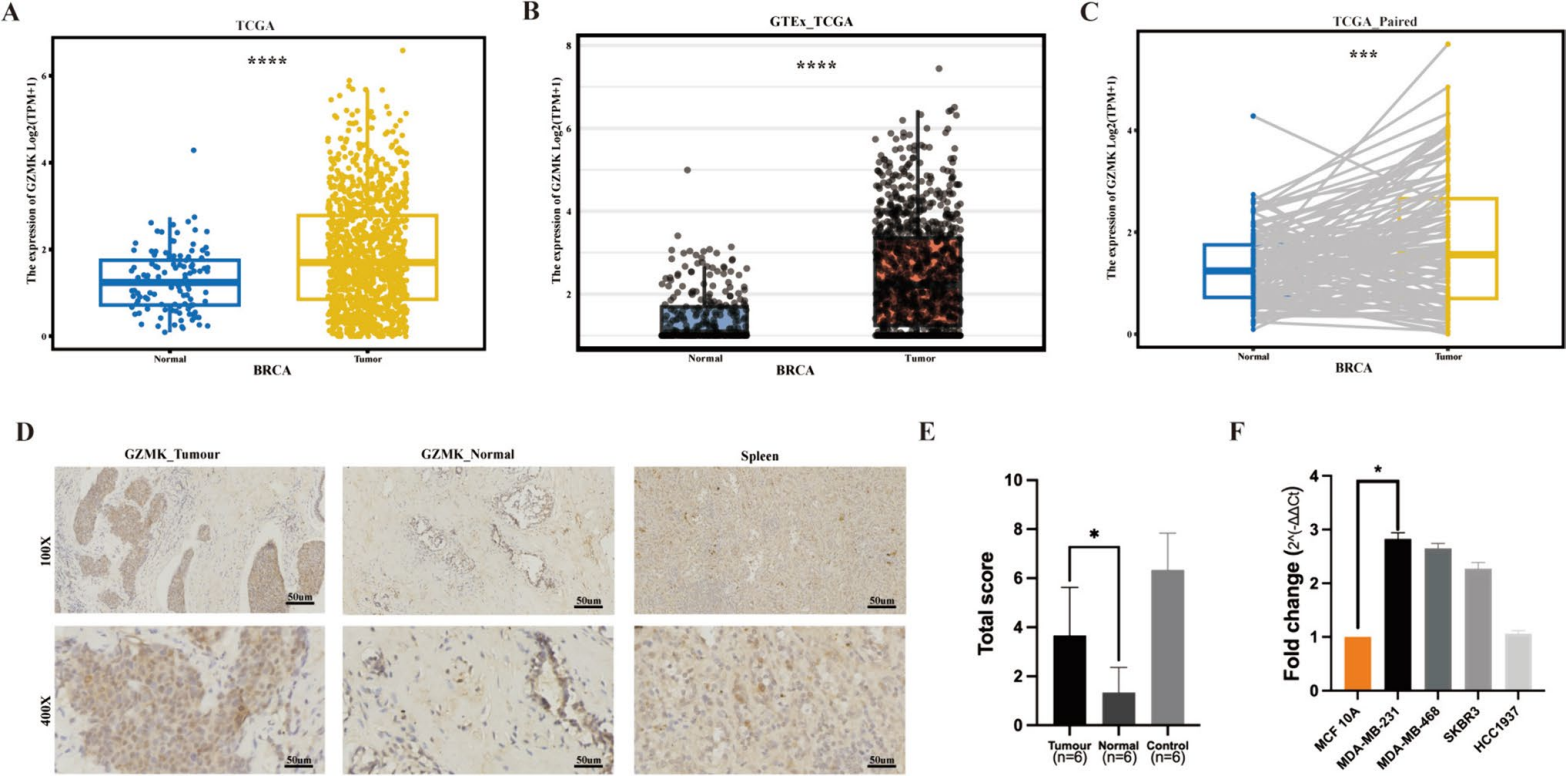
Illustrates the expression levels of GZMK in breast cancer tissues compared to normal tissues and across five cell lines. **A** Expression levels of GZMK in 1085 breast cancer tissues (BRCA) from the TCGA database compared to 112 normal tissues. **B** Analysis of GZMK expression levels in 1085 breast cancer tissues and 291 normal tissues from the GTEx database in conjunction with the TCGA database. **C** GZMK expression levels in 113 paired breast cancer tissues and normal tissues from the TCGA database. **D** Immunohistochemical results of GZMK expression in six breast cancer tissues and paired normal tissues, with splenic tissue used as positive control (*N* = 6). E Relative expression levels of GZMK in six breast cancer tissues, paired normal tissues, and six positive control splenic tissues. F PCR analysis of GZMK expression Ct values in five cell lines (*N* = 3). *P < 0.05, ***P < 0.001, ****P < 0.0001

An analysis of the TCGA database identified 3,559 DEGs and 4,894 OS-related DEGs, with an intersection of 245 genes (Figure S1A). Protein–protein interaction (PPI) analysis through the STRING database and Cytoscape software pinpointed the top 15 proteins based on interaction scores, including GZMK, highlighting its close association with patient survival (Figure S1B, C). We focused primarily on analyzing the expression levels of GZMK in breast cancer tissues and normal tissues using TCGA data, as well as in five cell lines. The results revealed higher expression levels in cancer tissues compared to normal tissues. Within the TCGA database, the relative expression levels of GZMK (log2(TPM + 1)) were analyzed in 1,085 BRCA and 112 normal tissue samples, showing significantly higher expression in cancer tissues (*P* < 0.0001) (Fig. 1A). Further analysis, which combined data from the GTEx and TCGA databases involving 1,085 breast cancer tissues and 291 normal tissues, also indicated significantly higher expression levels in cancer tissues (*P* < 0.0001) (Fig. 1B). Moreover, an analysis of 113 paired breast cancer and normal tissues from the TCGA database revealed significantly higher expression levels in cancer tissues (*P* < 0.0001) (Fig. 1C).

Additionally, six pairs of breast cancer tissues and matched normal epithelial tissues from Qinghai University Affiliated Hospital were analyzed. Immunohistochemistry analysis indicated significantly higher expression levels of GZMK in cancer tissues compared to normal tissues (*P* < 0.05) (Fig. 1D). A relative quantitative analysis of GZMK expression levels in these six pairs of tissues showed a mean expression level of 3.67 in cancer tissues compared to 1.33 in normal tissues, with a fold difference of 2.75 (*P* = 0.03, *P* < 0.05) (Fig. 1E). Furthermore, PCR analysis of GZMK Ct values in five cell lines revealed relatively higher expression levels in SKBR3, HCC1937, and MDA-MB-231 cell lines compared to MCF 10A (Fig. 1F).

### Relationship between GZMK expression levels and clinicopathological characteristics of breast cancer

We collected paraffin-embedded tissue sections from 131 diagnosed breast cancer patients at Qinghai University Affiliated Hospital between 2020 and 2023. These patients' clinicopathological characteristics were recorded, and none had received any treatment prior to specimen collection. The detailed basic information of the patients is listed in Table S2.

Immunohistochemistry was used to assess the relative expression levels of GZMK, which were then categorized into high and low expression groups based on the mean expression level. Statistical analysis showed significant differences in GZMK expression levels associated with tumor differentiation and lymph node staging (*P* < 0.05). However, no significant correlations were found with other clinicopathological characteristics, such as patient age, tumor side (left/right breast), tumor size, pathological staging, ER status, PR status, HER2 status, Ki-67 level, molecular subtype, androgen receptor status (AR), and vascular invasion (Table 1).

**Table 1:** Clinicopathological characteristics of breast cancer patients divided into High- and Low-GZMK expression groups

Characteristic	GZMK_Low, *N* = 80	GZMK_High, *N* = 51	*P* value
Patient age			0.723
< 54	45 (56%)	27 (53%)	
> = 54	35 (44%)	24 (47%)	
Location			0.859
Left	36 (45%)	24 (47%)	
Right	44 (55%)	27 (53%)	
Differentiation			0.03*
High	9 (11%)	0 (0%)	
Low	16 (20%)	13 (25%)	
Mediate	55 (69%)	38 (75%)	
Tumor			0.886
T1	36 (45%)	21 (41%)	
T2	39 (49%)	26 (51%)	
T3	5 (6.3%)	4 (7.8%)	
Positive lymph nodes			0.04*
N0	50 (63%)	20 (39%)	
N1	19 (24%)	23 (45%)	
N2	8 (10%)	5 (9.8%)	
N3	3 (3.8%)	3 (5.9%)	
Pathological staging			0.242
IA	25 (31%)	11 (22%)	
IIA	29 (36%)	14 (27%)	
IIB	15 (19%)	17 (33%)	
IIIA	8 (10%)	5 (9.8%)	
IIIC	3 (3.8%)	4 (7.8%)	
ER			0.364
Negative	13 (16%)	12 (24%)	
Positive	67 (84%)	39 (76%)	
PR			> 0.999
Negative	23 (29%)	15 (29%)	
Positive	57 (71%)	36 (71%)	
HER2			0.994
0	27 (34%)	16 (31%)	
1 +	26 (33%)	17 (33%)	
2 +	17 (21%)	12 (24%)	
3 +	10 (13%)	6 (12%)	
Ki67			0.593
< 20%	38 (48%)	27 (53%)	
> = 20%	42 (53%)	24 (47%)	
Molecular typing			0.552
HER-2_positive	4 (5.0%)	2 (3.9%)	
LuminalA	19 (24%)	15 (29%)	
LuminalB	48 (60%)	25 (49%)	
TNBC	9 (11%)	9 (18%)	
AR			0.322
Negative	10 (13%)	10 (20%)	
Positive	70 (88%)	41 (80%)	
Blood vessel			> 0.999
Negative	28 (35%)	17 (33%)	
Positive	52 (65%)	34 (67%)	

For continuous variables, comparison between groups was performed using *t* test, and for categorical variables, comparison between groups was performed using Fisher's exact probability test

*P < 0.05

Additionally, the relationship between mRNA relative expression levels of GZMK (log2(TPM + 1)) in the TCGA database and clinicopathological characteristics of patients was evaluated. Significant differences in GZMK expression were observed among molecular subtypes of breast cancer (*P* < 0.05). However, no statistical differences were noted concerning other clinical indicators, such as patient age, race, tumor size, pathological staging, ER status, PR status, and HER2 status (Table S1). Additional patient information is provided in Table S3.

Subgroup analysis revealed that GZMK expression levels were relatively higher in patients with ER-negative breast cancer (Figures S2A–D), PR-negative breast cancer (Figures S2F, G), and HER2-positive breast cancer (Figure S2E). Conversely, expression levels were lower in patients with metastatic breast cancer (M1) (Figure S2H). Additionally, patients who responded well to radiotherapy (Figure S2I), paclitaxel chemotherapy (Figure S2J), and other chemotherapies (Figure S2K) exhibited higher GZMK expression levels, with these differences reaching statistical significance (*P* < 0.05). In the analysis of molecular subtypes, it was found that the expression level of GZMK varies significantly among different subtypes (Figures S2L, M), with all differences being statistically significant (*P* < 0.05). Notably, GZMK expression levels were higher in patients under 50 years of age compared to older patients (Figure S2N), with this difference also statistically significant (*P* < 0.05). However, no significant differences in GZMK expression levels were observed across different stages of breast cancer (*P* > 0.05) (Figure S2O).

### 3.3 Construction, evaluation, and visualization of breast cancer OS prediction model based on multifactorial COX regression, and survival curves of OS, PFS, and RFS for GZMK high and low expression groups in breast cancer patients

**Fig. 2:**
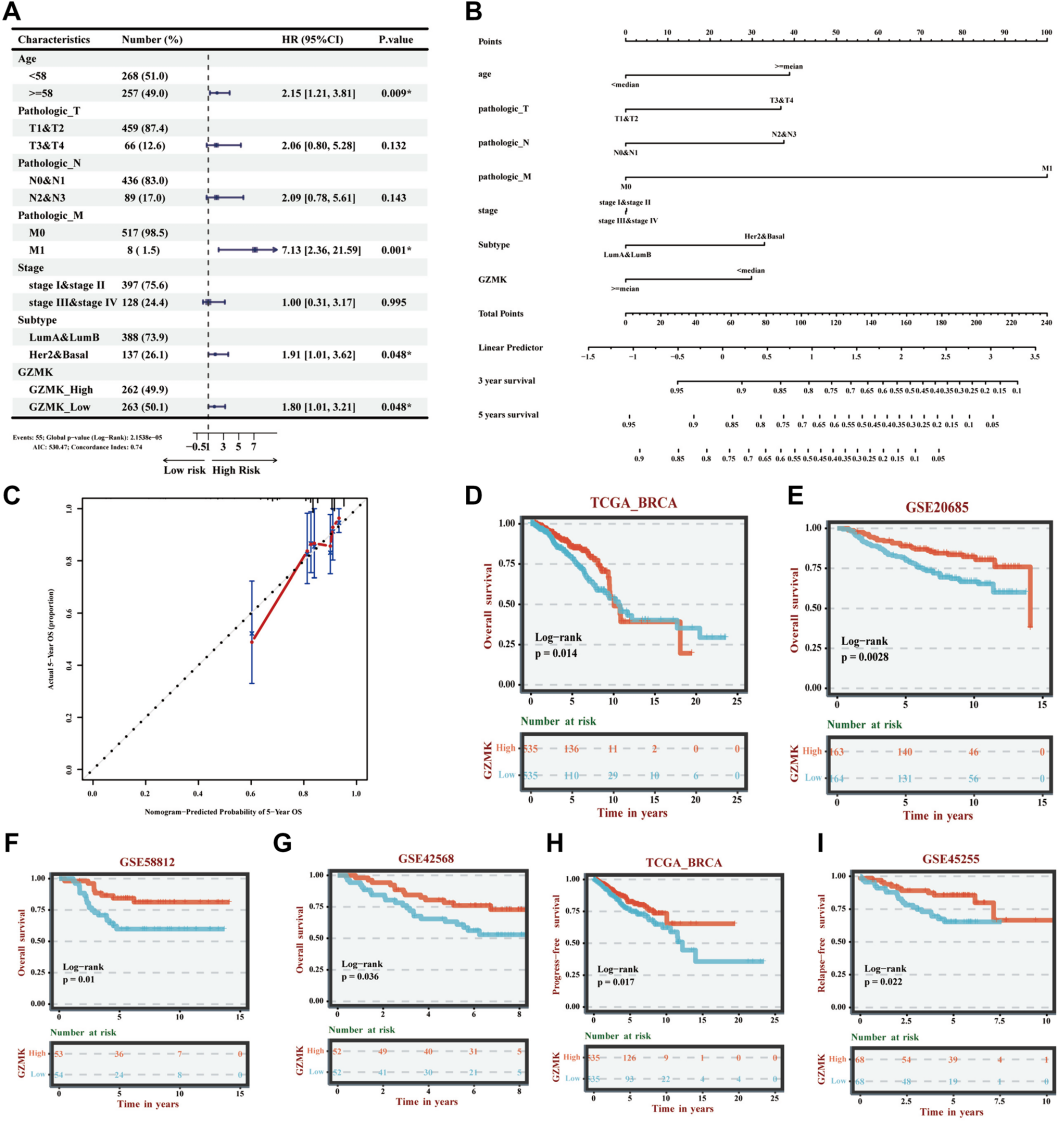
Depicts the construction of the OS prediction model for BRCA patients based on the TCGA database. **A** Forest plot of the results from the multivariable Cox regression analysis. **B** Visualization of the OS prediction model using a column line chart. **C** Calibration plot assessing the accuracy of the OS prediction model. **D** Kaplan–Meier (KM) survival curves for OS of the high and low GZMK expression groups in the TCGA database. **E** KM survival curves for OS of the high and low GZMK expression groups in the GSE20685 dataset. **F** KM survival curves for OS of the high and low GZMK expression groups in the GSE58812 dataset. **G** KM survival curves for OS of the high and low GZMK expression groups in the GSE42568 dataset. **H** KM survival curves for OS of the high and low GZMK expression groups in the TCGA database with PRS. **I** KM survival curves for RFS of the high and low GZMK expression groups in the GSE45255 dataset. *P < 0.05

A multifactorial COX regression analysis was performed to assess the impact of clinical-pathological indicators (age, pathological T stage, N stage, M stage, overall stage, and molecular subtype) and GZMK expression levels on the OS of breast cancer patients in the TCGA database. The analysis identified that patient age, M1 stage, HER2 and Basal subtypes, and low GZMK expression (GZMK_Low) were associated with unfavorable OS outcomes (Fig. 2A). The results of the multifactorial COX regression model were visualized using a nomogram (Fig. 2B), and the predictive accuracy of the model for OS was evaluated through a calibration curve (Fig. 2C).

**Fig. 3:**
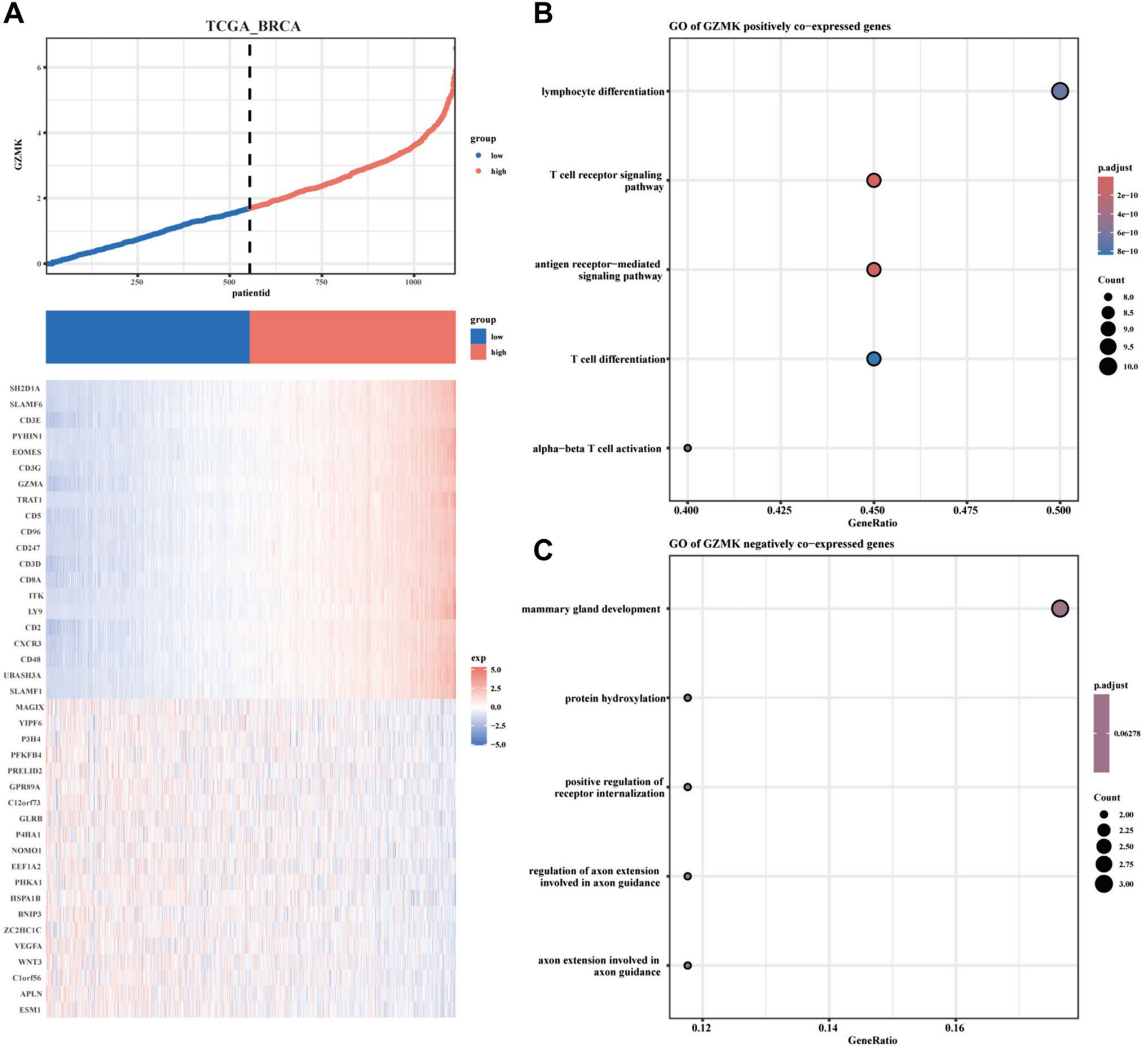
Presents the analysis of breast cancer patients from the TCGA database, including the top 20 most correlated genes with high and low expression of GZMK, as well as the main molecular functions and signaling pathways from the Gene Ontology (GO) analysis. **A** Heatmap of the top 20 most correlated genes with high and low expression of GZMK. **B** Bubble plot of the GO analysis showing the main molecular functions and signaling pathways positively correlated with GZMK expression. **C** Bubble plot of the GO analysis showing the main molecular functions and signaling pathways negatively correlated with GZMK expression

Furthermore, the impact of high and low GZMK expression groups on OS, progression-free survival (PFS), and recurrence-free survival (RFS) was analyzed. Survival curve analysis showed that high GZMK expression significantly improved OS, PFS, and RFS in patients. Specifically, in the TCGA database, the analysis included 1,070 breast cancer patients, with 535 in each expression group. High GZMK expression was associated with significantly improved OS (*P* < 0.05) (Fig. 2D). This finding was corroborated by the analysis of three GEO datasets (GSE20685, GSE58812, and GSE42568), which also indicated significant improvement in OS for patients with high GZMK expression (Figs. 2E–G). Additionally, in the TCGA database, high GZMK expression was linked to improved PFS (*P* < 0.05) (Fig. 3H). Analysis of the GEO dataset GSE45255 revealed that high GZMK expression significantly enhanced RFS, with statistically significant differences (*P* < 0.05) (Fig. 2I).

Further analysis using the Kaplan–Meier Plotter database demonstrated the impact of GZMK on breast cancer patient OS. Among all breast cancer patients (*n* = 1,879), those in the high GZMK expression group (*n* = 935) had significantly better OS compared to those in the low expression group (*n* = 944), with statistical significance (HR = 0.58 [0.48–0.71], *P* < 0.001) (Figure S3A). Subgroup analysis across different breast cancer subtypes showed that high GZMK expression improved OS in triple-negative breast cancer (TNBC) (n = 404), Luminal A (*n* = 794), Luminal B (*n* = 515), and HER2-positive breast cancer (*n* = 166), with statistically significant differences (*P* < 0.05) (Figures S3B–E).

The impact of GZMK on breast cancer patient RFS was similarly analyzed using the Kaplan–Meier Plotter database. Among all breast cancer patients (*n* = 4929), the RFS of the high GZMK expression group (*n* = 2464) was superior to that of the low expression group (*n* = 2465), with statistical significance (HR = 0.78 [0.71–0.87], *P* < 0.001) (Figure S4A). Subgroup analysis in different breast cancer subtypes revealed thathigh GZMK expression could improve RFS in TNBC, Luminal A, Luminal B, and HER2-positive breast cancer, with statistically significant differences (*P* < 0.05) (Figures S4B–E).

### Analysis of genes associated with GZMK high and low expression groups and GO /KEGG analysis

Further analysis was conducted on the relative mRNA sequencing expression levels (log2(TPM + 1)) of the top 20 genes most correlated with high and low GZMK expression groups in breast cancer patients from the TCGA database. A heatmap was generated for visualization. It was found that the high GZMK expression group was closely correlated with genes such as SH2D1A, SLAMF6, CD3E, PYHIN1, BOMES, CD3G, GZMA, TRAT1, CD5, CD96, CD247, CD3D, ITK, LY9, CD2, CXCR3, CD48, and SLAMF1. In contrast, the low GZMK expression group was closely correlated with genes such as MAGIX, YIPF6, P3H4, PFKFRA, PRELID2, GPR89A, C12orf73, GLRB, P4HA1, NOMO1, REF1A2, PHKA1, HSPAIR, BNIP3, VEGEA, WNT3, Clorf56, and ESMI (Fig. 3A).

Additionally, GO analysis was carried out to explore the molecular functions and signaling pathways primarily associated with genes positively correlated with GZMK expression. The main molecular functions included lymphocyte differentiation, T-cell differentiation, and alpha–beta T-cell activation. The principal signaling pathways were the T-cell receptor signaling pathway and antigen receptor-mediated signaling pathway (Fig. 3B). Conversely, genes negatively correlated with GZMK expression were primarily associated with molecular functions such as mammary gland development, protein hydroxylation, positive regulation of receptor internalization, and regulation of axon extension involved in axon guidance (Fig. 3C).

Intersection analysis of differentially expressed genes and survival-associated genes (245 genes) from the TCGA database, using GO, KEGG, and GSVA analysis, identified several biological processes. These processes included T-cell activity, regulation of T-cell activity, lymphocyte adhesion, monocyte differentiation, antigen receptor regulatory signaling pathways, regulation of lymphocyte adhesion, lymphocyte differentiation, T-cell receptor signaling pathway, T-cell differentiation, and positive regulation of T-cell activity, which were mainly involved in regulating T-cell activity (Figure S5A). Furthermore, 15 major signaling pathways were identified, with the T-cell receptor signaling pathway being the primary one, closely related to pathways such as PD-L1 expression and PD-1 checkpoint pathways in tumors, NF-κB signaling pathway, and natural killer cell-mediated cytotoxicity (Figure S5B). GSVA analysis revealed 49 gene-related pathways, with 36 activated pathways, 4 pathways showing no activity changes, and 9 inhibited pathways. Key activated pathways included IL6_JAK_STAT3_SIGNALING, while pathways like ESTROGEN_RESPONSE_LATE were inhibited (Figure S5C).

**Fig. 4:**
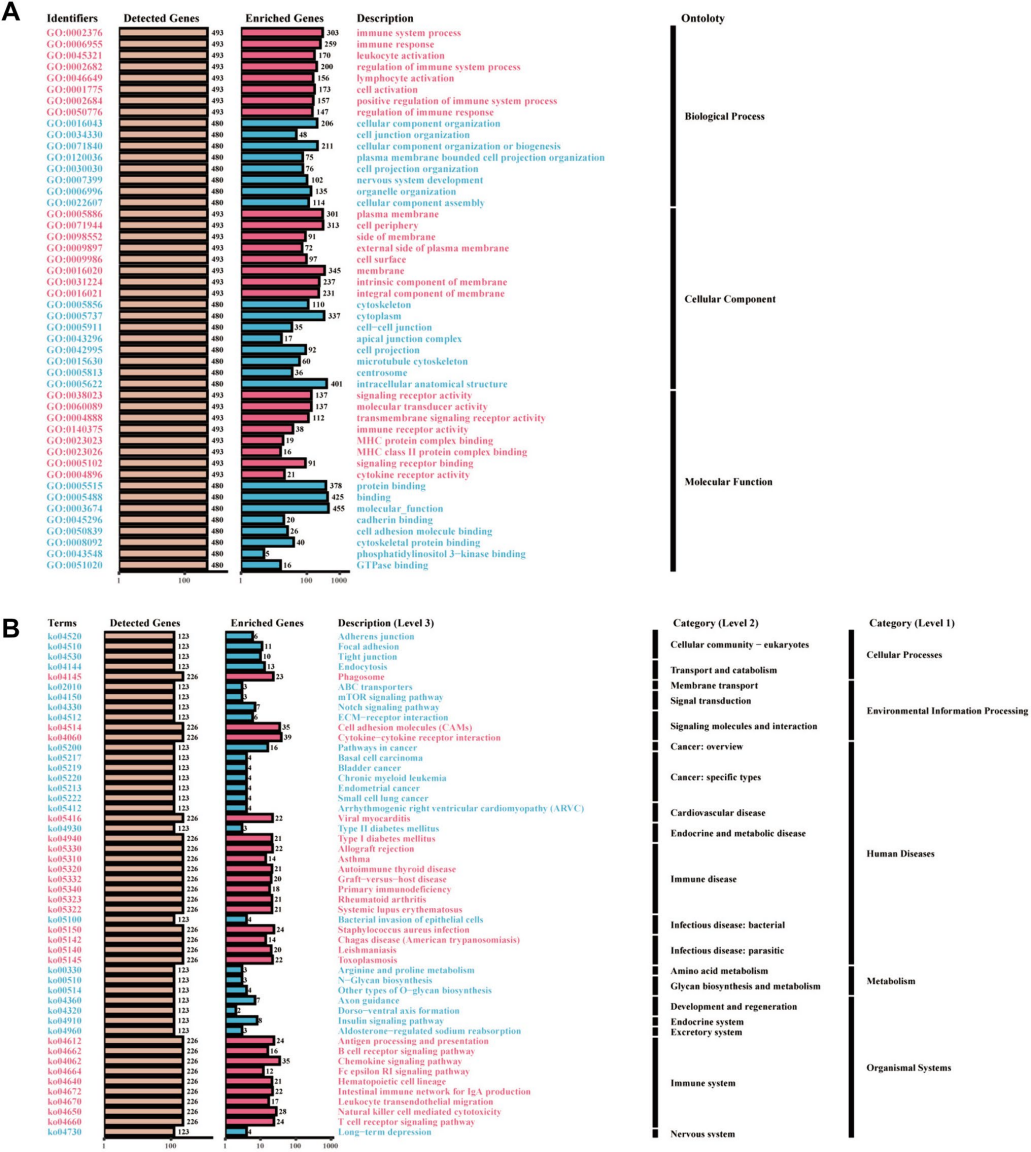
Depicts the Gene Ontology (GO) and Kyoto Encyclopedia of Genes and Genomes (KEGG) bar plots resulting from the overrepresentation analysis (ORA) of GZMK in The Cancer Genome Atlas (TCGA) database and 19 Gene Expression Omnibus (GEO) datasets (GSE11121, GSE12093, GSE162228, GSE17705, GSE20685, GSE20711, GSE21653, GSE22219, GSE25055, GSE25065, GSE42568, GSE45255, GSE48390, GSE58812, GSE61304, GSE7390, GSE88770, GSE97342, and GSE9893). **A** GO bar plot. **B** KEGG bar plot

Integrated analysis of the TCGA database and 19 GEO datasets (GSE11121, GSE12093, GSE162228, GSE17705, GSE20685, GSE20711, GSE21653, GSE22219, GSE25055, GSE25065, GSE42568, GSE45255, GSE48390, GSE58812, GSE61304, GSE7390, GSE88770, GSE97342, and GSE9893) for GZMK overexpression analysis (ORA) revealed GO and KEGG results, indicating biological processes primarily associated with the immune system process and immune response, and molecular functions associated with immune receptor activity and MHC protein complex binding (Fig. 4A). KEGG results showed a close relationship with the immune system, primarily involving pathways such as antigen processing and presentation, B cell receptor signaling pathway, natural killer cell-mediated cytotoxicity, and T cell receptor signaling pathway (Fig. 4B). GSEA enrichment analysis revealed similar results, primarily associated with adaptive immune response, lymphocyte-mediated immunity, positive regulation of leukocyte cell adhesion, and inflammatory response (Figure S6A, B, C). Analysis of the TCGA database alone also suggested a close association with autoimmune thyroid disease (Figure S6D, E).

### Analysis of GZMK in the tumor microenvironment (TME) of breast cancer: immune cell infiltration

**Fig. 5:**
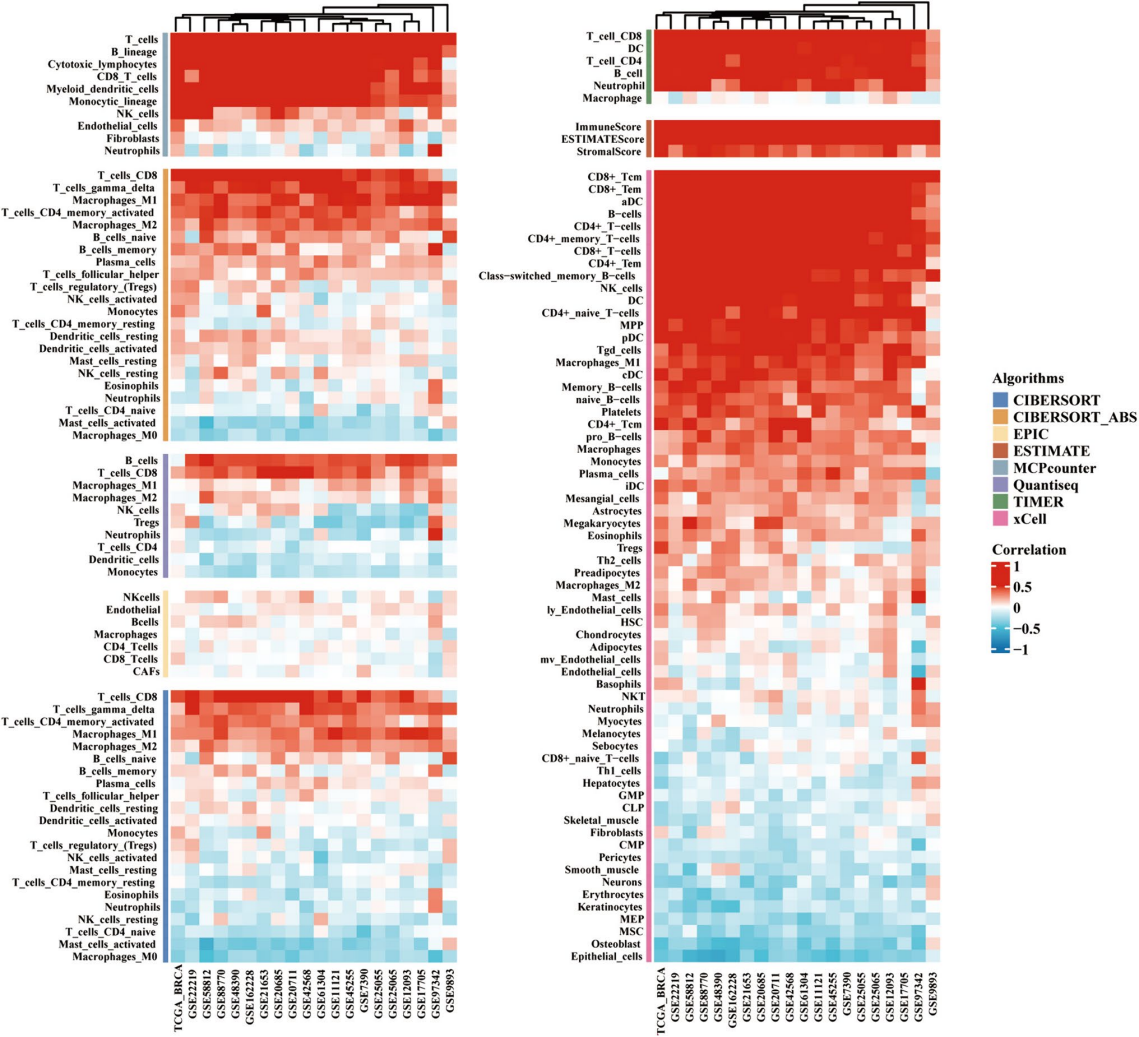
Illustrates the evaluation of GZMK in the tumor microenvironment (TME) of breast cancer using eight common algorithms to assess immune cell infiltration

GZMK's role in the tumor microenvironment (TME) of breast cancer was assessed based on the eight most common algorithms (CIBERSORT, CIBERSORT ABS, EPIC, ESTIMATE, MCP-counter, Quantiseq, TIMER, and xCell) (Fig. 5). Analysis of both TCGA database and 19 GEO datasets (GSE11121, GSE12093, GSE162228, GSE17705, GSE20685, GSE20711, GSE21653, GSE22219, GSE25055, GSE25065, GSE42568, GSE45255, GSE48390, GSE58812, GSE61304, GSE7390, GSE88770, GSE97342, and GSE9893) revealed heatmap results of the eight algorithms, indicating that GZMK was most correlated with CD8^+^ T cells. While the Quantiseq algorithm ranked B cells as the most correlated, the remaining seven algorithms consistently ranked CD8^+^ T cells as the top correlation (Fig. 5).

Analysis of the TCGA database revealed a strong correlation between GZMK expression levels and major immune indicators. GZMK expression levels were highly correlated with ESTIMATE Score_estimate (Cor = 0.743, *P* < 0.001), ImmuneScore_estimate (Cor = 0.802, *P* < 0.001), and StromalScore_estimate (Cor = 0.516, *P* < 0.001). Additionally, using the MCP-counter algorithm, GZMK expression levels showed a high correlation with CD8_T_cells (Cor = 0.825, *P* < 0.001). Similar correlations were observed using the xCell algorithm (Cor = 0.843, *P* < 0.001) and the TIMER algorithm (Cor = 0.782, *P* < 0.001) (Figure S7A-F).

### Correlation analysis of GZMK with antigen presentation-related genes, immune checkpoint genes, immune enhancer genes, chemokines, and receptors in breast cancer

**Fig. 6:**
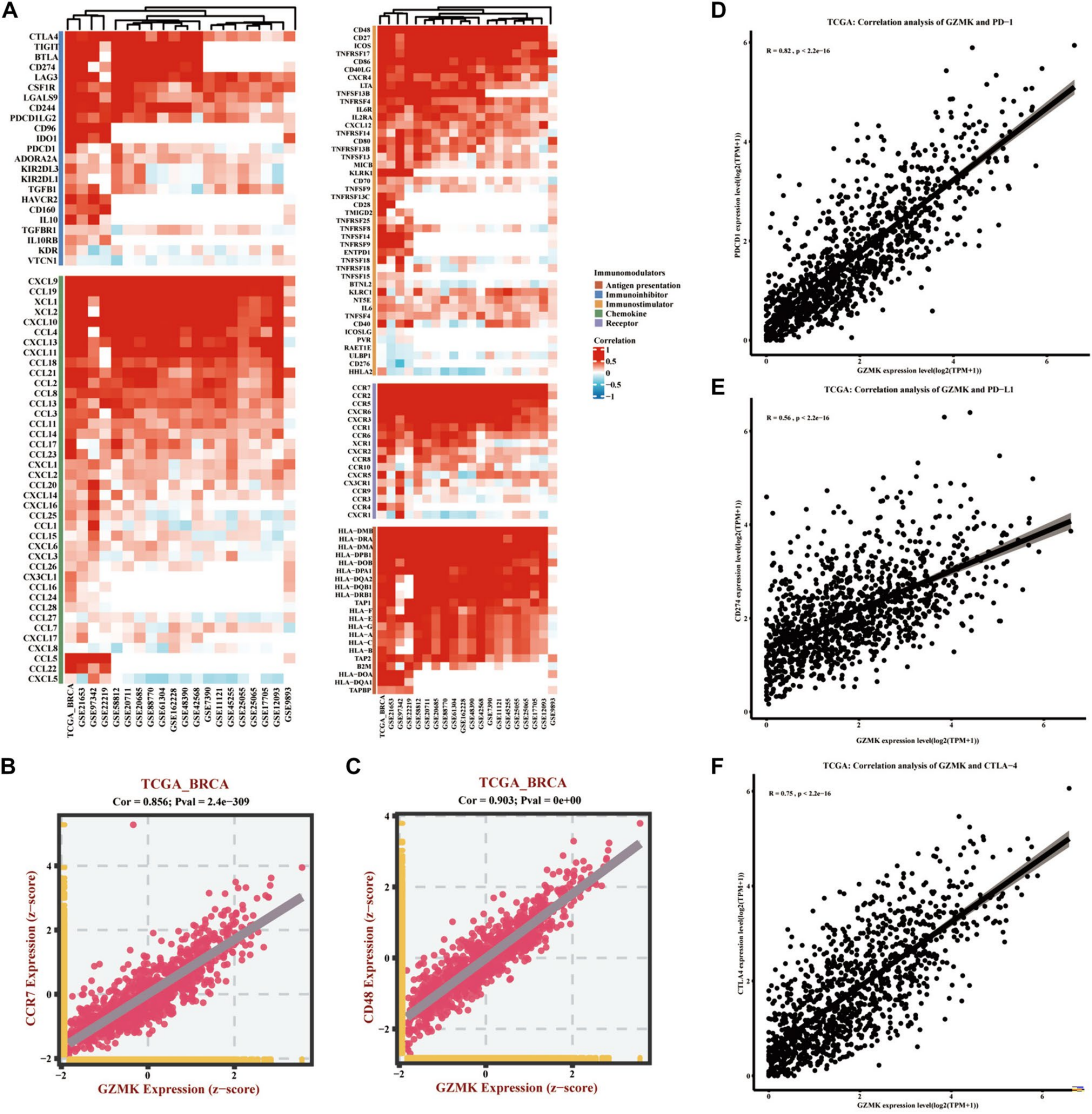
Depicts the analysis of the correlation between GZMK and five types of immune modulators (antigen presentation, immune inhibitors, immune enhancers, chemokines and receptors) in breast cancer. **A** Heatmap of the correlation analysis between GZMK and these immune modulators in TCGA database and 19 GEO datasets. **B** Scatter plot showing the correlation between GZMK and CCR7 in TCGA database. **C** Scatter plot showing the correlation between GZMK and CD48 in TCGA database. **D** Scatter plot showing the correlation between GZMK and PD-1 in TCGA database. **E** Scatter plot showing the correlation between GZMK and PD-L1 in TCGA database. **F** Scatter plot showing the correlation between GZMK and CTLA4 in TCGA database

Analysis of the correlation between GZMK and antigen presentation-related genes, immune checkpoint genes, immune enhancer genes, chemokines, and receptors was conducted using the TCGA database and 19 GEO datasets (GSE11121, GSE12093, GSE162228, GSE17705, GSE20685, GSE20711, GSE21653, GSE22219, GSE25055, GSE25065, GSE42568, GSE45255, GSE48390, GSE58812, GSE61304, GSE7390, GSE88770, GSE97342, and GSE9893). The summarized results are presented in a heatmap (Fig. 6A). Notably, GZMK showed the strongest correlations with HLA-DMB (antigen presentation-related gene), CTLA4 (immune checkpoint gene), CD48 (immune enhancer gene), CXCL9 (chemokine-related gene), and CCR7 (receptor-related gene) (Fig. 6A).

In an analysis of the TCGA database, mRNA relative expression levels of GZMK (log2(TPM + 1)) were correlated with CTLA4, PD-1, PD-L1, CD48, and CCR7 in patients with breast cancer. The results demonstrated strong correlations, with the correlation coefficient between GZMK and CCR7 at 0.856 (*P* < 0.001) (Fig. 6B), between GZMK and CD48 at 0.903 (*P* < 0.001) (Fig. 6C), between GZMK and PD-1 at 0.82 (*P* < 0.001) (Fig. 6D), between GZMK and PD-L1 at 0.56 (*P* < 0.001) (Fig. 6E), and between GZMK and CTLA4 at 0.75 (*P* < 0.001) (Fig. 6F).

### Analysis of GZMK in predicting immunotherapeutic efficacy

**Fig. 7:**
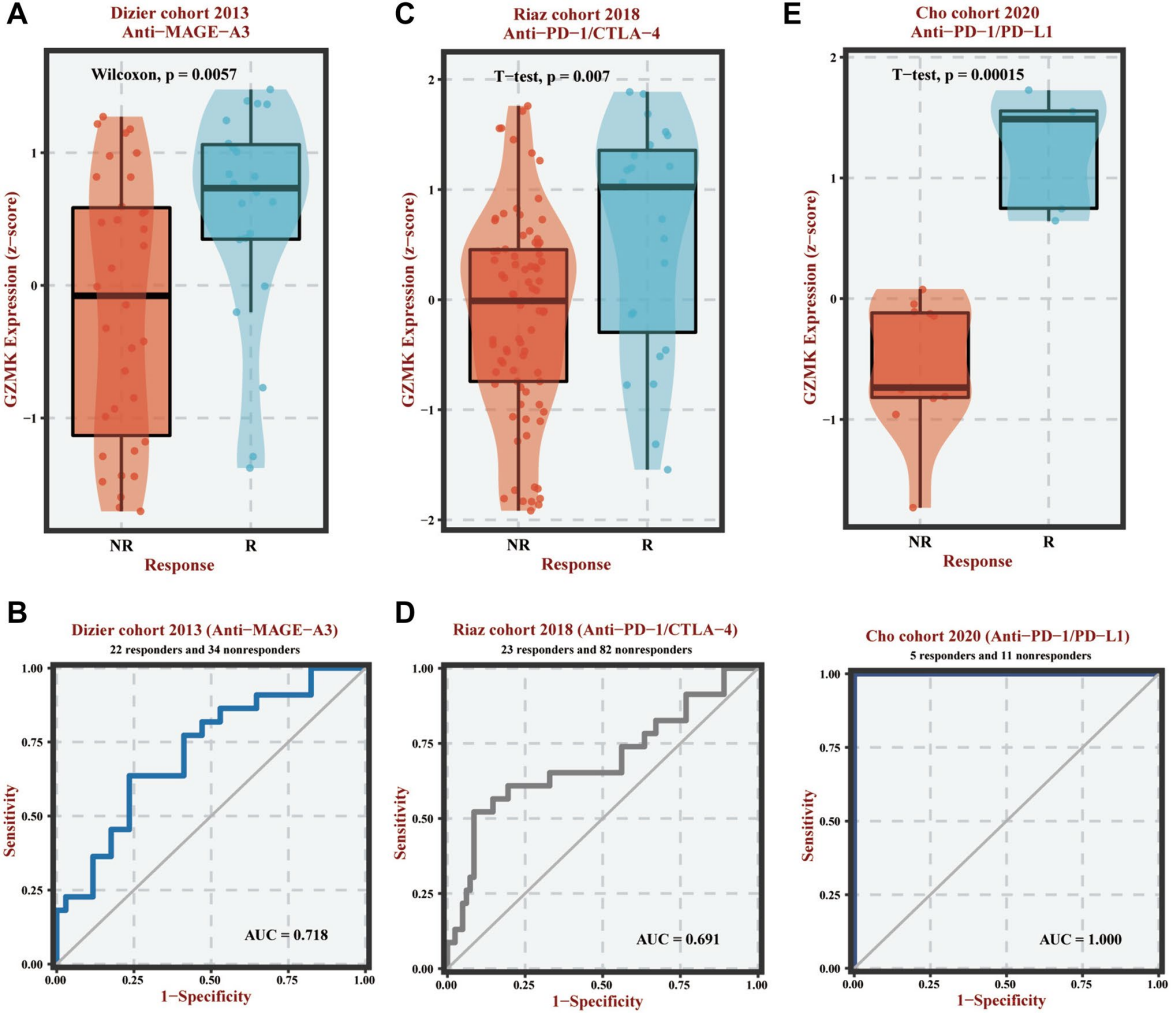
Illustrates the analysis of GZMK in predicting immune therapy efficacy. **A**, **C**, **E** Box plots visualize the differential expression of GZMK between the responsive and non-responsive groups. **B**, **D**, **F** Receiver operating characteristic (ROC) curves evaluate the performance of GZMK in predicting immune therapy efficacy

Our research identified three significant findings that suggest high expression of GZMK can enhance a patient's responsiveness to immunotherapy (Fig. 7). In a study focusing on MAGE-A3 antibody immunotherapy, a comparative analysis of tissue microarray data from 22 melanoma patients who responded to immunotherapy and 34 who progressed showed significantly higher GZMK expression levels in the responders (*P* < 0.05) (Fig. 7A). Additionally, GZMK's predictive accuracy for MAGE-A3 antibody efficacy was demonstrated with an AUC of 0.718 (Fig. 7B).

In the examination of transcriptome sequencing data before and after treatment with nivolumab (PD-1/CTLA-4 antibodies) in 105 advanced melanoma patients, those who responded to the therapy (23 cases) showed significantly higher GZMK expression (*P* < 0.05) (Fig. 7C). The predictive accuracy of GZMK for PD-1/CTLA-4 antibody efficacy was also notable, with an AUC of 0.691 (Fig. 7D).

Moreover, analysis of transcriptome sequencing data from 16 non-small cell lung cancer patients treated with nivolumab or pembrolizumab (PD-1 antibodies) revealed that, among these patients, five experienced disease remission while eleven did not. Higher GZMK expression was observed in the responders, with statistically significant differences (*P* < 0.05) (Fig. 7E). Additionally, the predictive accuracy of GZMK for PD-1 antibody efficacy was found to be exceptionally high, approaching 1 (Fig. 6F). GZMK was also found to increase sensitivity to tamoxifen (Figure S8A, B, C) and enhance resistance to platinum-based drugs (Figure S8D, E, F).

### Functional validation of GZMK in MDA-MB-231 breast cancer cell line

**Fig. 8:**
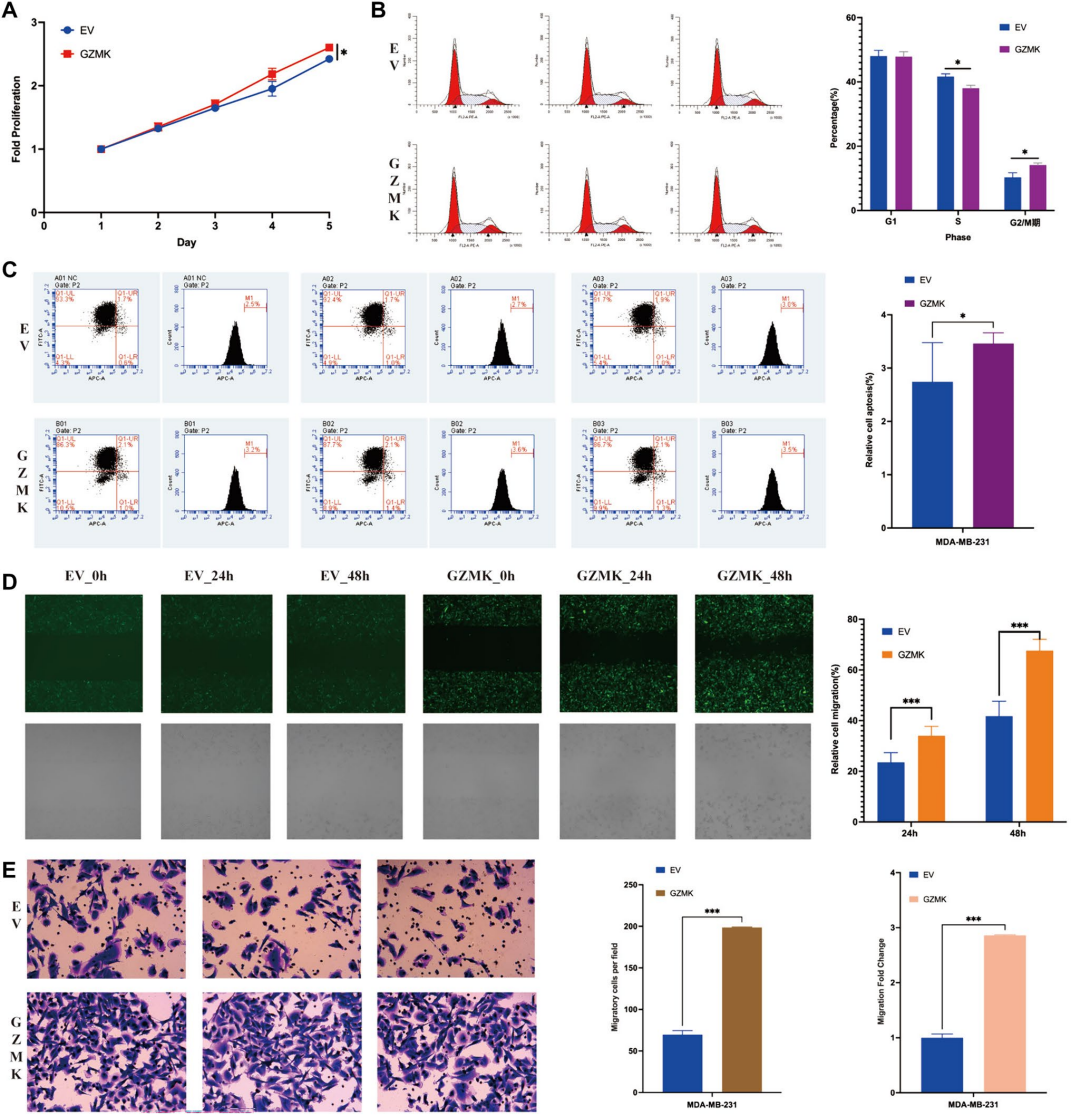
Illustrates the effects of GZMK overexpression on MDA-MB-231 breast cancer cells in vitro. **A** Cell proliferation activity detected by the CCK-8 assay, showing increased proliferation capacity in the GZMK overexpression group. **B** Results of cell cycle experiments indicating an increase in the G2/M phase in the GZMK overexpression group. **C** Results of apoptosis experiments showing an increase in apoptosis in the GZMK overexpression group. **D** Results of Celigo scratch assay indicating increased migration capability of tumor cells in the GZMK overexpression group. **E** Results of Transwell invasion assay showing increased invasion capability of tumor cells in the GZMK overexpression group. *P < 0.05; ***P < 0.001

We conducted cellular functional experiments to analyze the effects of GZMK overexpression in MDA-MB-231 cells (Fig. 8). The results were as follows:

Cell Proliferation: CCK-8 assay results showed that, compared to the control group (NC), the GZMK-overexpressing group demonstrated increased cell proliferation on Day 5, with a significant difference (*P* < 0.05) and inter-group measurement differences of less than 20%, suggesting enhanced proliferation capacity (Fig. 8A).

Cell Cycle: Analysis revealed no significant difference in the G1 phase between the groups (*P* > 0.05), but significant increases in the S and G2/M phases were observed in the GZMK group (*P* < 0.05), indicating promotion of cell division (Fig. 8B).

Apoptosis: Apoptosis assays indicated that the GZMK group had a higher rate of apoptosis compared to the NC group, with both groups showing rates below 5% (*P* < 0.05), suggesting that GZMK promotes apoptosis (Fig. 8C).

Scratch Test (Celigo): The scratch test revealed a significantly higher cell migration rate in the GZMK group at 48 h compared to the NC group (*P* < 0.05), indicating enhanced migration ability (Fig. 8D).

Cell Invasion (Transwell Assay): The Transwell assay showed that the GZMK group had a significantly higher invasion transfer rate than the NC group (*P* < 0.05), indicating increased invasion capacity (Fig. 8E).

## Discussion

Little is known about the molecular function of GZMK, a trypsin-like serine protease in the granule enzyme family alongside its homolog, GZMA (Bovenschen and Kummer 2010). Like trypsin, GZMK exhibits activity upon alkalization with arginine or lysine (Bovenschen et al. 2009). Human GZMK was first discovered in 1988 after being purified from human peripheral blood mononuclear cells (Bade et al. 2005). Due to their similar characteristics as serine proteases and common substrates, GZMK has often been considered a "backup enzyme" for GZMA. However, this concept is currently under debate due to the unique structure and function of GZMK (Bovenschen et al. 2009; Kurschus et al. 2005; Jenkins et al. 2008).

Several intracellular substrates of GZMK, including SET, Bid, Ape1, VCP, and p53 (Zhao et al. 2007a, 2007b; Guo et al. 2008, 2010; Hirata et al. 2014), have been identified. These substrates contribute to cell toxicity, mitochondrial damage, and ROS accumulation through caspase-independent pathways, playing significant roles in tumors and inflammation (Bouwman et al. 2021). GZMK protein has been detected in human colorectal tumor tissues, showing a positive correlation with sVEGFR1 protein levels and a negative correlation with tumor angiogenesis and size in T4-stage tumors (Tiberti et al. 2022). Our study also reveals differential expression of GZMK in breast cancer, with higher expression levels noted in cancer tissues (Fig. 1). Immunohistochemical analysis and evaluation of GZMK expression levels in cell lines, combined with bioinformatics analysis, corroborate our research findings. Subgroup analysis further reveals higher expression of GZMK in hormone receptor-negative and HER2-positive patients (Figure S2). Clinical pathological feature analysis demonstrates a close correlation between GZMK and lymph node staging, degree of differentiation (Table 1), and molecular subtypes (Table S2).

When analyzing the impact of GZMK on the survival of breast cancer patients, it was found that high expression of GZMK can improve patients' OS, PFS, RFS (Fig. 2D–I). A specific analysis of the impact of GZMK high and low expression groups on OS in the TCGA database revealed a total of 1070 cases of breast cancer patients, with 535 patients in each group. The high expression group of GZMK significantly improved the OS of patients, with a statistically significant difference (*P* = 0.014) (Fig. 2D). Analysis of three GEO datasets (GSE20685, GSE58812, and GSE42568) also revealed that the high expression group of GZMK can significantly improve patients' OS, with statistically significant differences (*P* < 0.05) (Fig. 2E, F, G). Further validation through the Kaplan–Meier Plotter database (Figure S3–4) showed that the high expression group of GZMK can improve patient OS in all four subtypes (Figure S3A–E). In all breast cancer patients (*n* = 1879 cases), the high expression group of GZMK (*n* = 935 cases) had better OS than the low expression group (*n* = 944 cases), with statistical significance (HR = 0.58 (0.48–0.71), *P* < 0.001) (Figure S3A). Similar findings were observed in the analysis of different subtypes of breast cancer (Figure S3). Analysis of the impact of GZMK on RFS in breast cancer patients showed that in all breast cancer patients (*n* = 4929 cases), the high expression group of GZMK (*n* = 2464 cases) had better RFS than the low expression group (*n* = 2465 cases), with statistical significance (HR = 0.78 (0.71–0.87), *P* < 0.001) (Figure S4A); similar findings were observed in the analysis of different subtypes of breast cancer (Figure S4).

In studies related to GZMA, it was similarly found that high expression of GZMA enhanced patients' OS, disease-specific survival (DSS), and progression-free interval (PFI). Based on multivariable Cox regression analysis, GZMA was identified as an independent favorable prognostic factor for breast cancer (Huo et al. 2023). In studies related to GZMB, it was observed that in the PD-L1-positive group, Kaplan–Meier analysis revealed better recurrence-free survival (RFS) and OS in triple-negative breast cancer (TNBC) patients with high expression of GZMB (defined as ≥ 1% tumor-infiltrating lymphocytes (TILs) positive). Both RFS and OS were significantly prolonged in patients with high GZMB expression (RFS: *P* = 0.0220, OS: *P* = 0.0254) (Mizoguchi et al. 2023). Multivariable Cox analysis also indicated better OS in patients with high expressions of PD-L1 and GZMB (hazard ratio: 0.25 (95% CI: 0.07–0.88), *P* = 0.03), suggesting that GZMB is a useful prognostic biomarker for PD-L1-positive TNBC patients (Mizoguchi et al. 2023). Additionally, a study found that high expression of GZMK in advanced melanoma significantly improved patients' PFS and OS (PFS: *P* = 0.04, OS: *P* = 0.01) (Wu et al. 2021b). Another study developed a transcriptomic signature from eight immune-related genes (BLK, GZMM, CXCR6, LILRA1, SPIB, CCL4, CXCR4, SLAMF7) that could predict recurrence in the cohort. The high-expression group showed improved disease-free recurrence interval (DRFI) (HR: 0.18 (95% CI: 0.09–0.35), *P* < 0.001), RFS (HR: 0.54 (95% CI: 0.4–0.74), *P* < 0.001), and disease-specific survival (DSS) (HR: 0.53 (95% CI: 0.35–0.8), *P* = 0.002) in triple-negative breast cancer patients with residual disease after neoadjuvant chemotherapy (Blaye et al. 2022).

The analysis reveals the primary molecular functions and signaling pathways associated with genes co-expressed with GZMK. The key molecular functions include lymphocyte differentiation, T cell differentiation, and alpha-beta T cell activation. The principal signaling pathways are the T cell receptor signaling pathway and the antigen receptor-mediated signaling pathway (Fig. 3B). Using Overrepresentation Analysis (ORA) along with GO and KEGG pathway enrichment analyses, we have identified a close association with immune-related pathways (Fig. 4A, B). Understanding the molecular dynamics of T cell differentiation advances our knowledge of T cell biology and opens new possibilities for clinical applications. In a retrospective analysis of non-small cell lung cancer (NSCLC) patients undergoing anti-PD-1/PD-L1 therapy, those with higher CD5 expression on their effector memory T cells (Tem) showed improved responses compared to those with lower expression (Kim et al. 2023). Studies also show that Treg cells inhibit the activity of NK and CD8^+^ T cells through the GZMB-perforin pathway, and GZMB-deficient mice are more effective in clearing both allogeneic and syngeneic tumor cell lines (Cao et al. 2007). Our research identifies GZMK as a regulatory factor in T cell differentiation and highlights it as a new target for enhancing immunotherapy.

Over the past 20 years, significant advancements in tumor immunology have established immunotherapy as a major breakthrough in oncology (Agache et al. 2022; Billan et al. 2020). Research has confirmed that numerous interconnected factors influence anti-tumor immune responses. Each of these factors, alone or in combination, can serve as targets for cancer immunotherapy (Wu et al. 2022). Currently, immune checkpoint inhibitors, especially those targeting PD-1 or its ligand PD-L1, are increasingly explored as potential therapeutic strategies for various cancers (Topalian et al. 2020). In the Impassion130 biomarker subgroup analysis, the expression of PD-L1 on immune cells is positively correlated with the number of CD8^+^ T cells. High levels of both are associated with prolonged PFS and OS in breast cancer patients (Emens et al. 2021). However, the response rates of many TNBC patients to PD-1/PD-L1 inhibitors as monotherapy are not high, highlighting the clinical need to identify other immune checkpoint inhibitors (ICIs) that act synergistically with PD-1/PD-L1 inhibitors to improve efficacy when used in combination (Wu et al. 2022; Ohaegbulam et al. 2015; Doroshow et al. 2021; Zhang et al. 2021).

In our analysis of GZMK and immune cell infiltration in the TME of breast cancer, we found a close relationship with T cells. We also observed strong correlations with ESTIMATEScore_estimate (Cor = 0.743, *P* < 0.001), ImmuneScore_estimate (Cor = 0.802, *P* < 0.001), and StromalScore_estimate (Cor = 0.516, *P* < 0.001) (Figure S7A, B, C). In the correlation analysis of GZMK with genes related to antigen presentation, immune checkpoints, immune inhibitors, chemokines, and their receptors, we found strong correlations with CTLA4 (Cor = 0.856, *P* < 0.001), PD-1 (Cor = 0.82, *P* < 0.001), PD-L1 (Cor = 0.56, *P* < 0.001), CD48 (Cor = 0.75, *P* < 0.001), and CCR7 (Cor = 0.856, *P* < 0.001) (Fig. 6B–F). There is a significant correlation between GZMK and PD-1/PD-L1 (Fig. 6D, E), providing new possibilities for mechanistic research in breast cancer immunotherapy. Through this association, it may be possible to discover new breakthroughs in the study of GZMK as a target in immunotherapy for triple-negative breast cancer.

Studies have indicated that the granzyme family acts as predictive biomarkers for cutaneous melanoma (CM), with the high-expression group benefiting more from anti-PD-1 immunotherapy. It has also been found that GZMK plays an anti-cancer role in CM, activating immune signaling pathways and responses in low-risk patients, especially when CD8^+^ T cells are highly expressed, resulting in greater benefits from anti-PD-1 therapy (Wu et al. 2021b). Similarly, our research has found that GZMK is involvedin the activation of the PD-L1 and PD-1 checkpoint pathways in tumors (Figure S5A). This suggests that GZMK-positive breast cancer may benefit more from anti-PD-1/PD-L1 immunotherapy, indicating that GZMK could be a target for predicting the efficacy of immunotherapy in breast cancer.

Immune checkpoint molecules represent one of the most promising targets for cancer therapy. Antibodies that target PD-1/PD-L1 are extensively utilized in clinical practice and hold significant potential (Teng et al. 2018). However, challenges such as low response rates in certain cancers (e.g., melanoma, breast cancer, and lung cancer), a lack of well-defined biomarkers, immune-related toxicities, and intrinsic or acquired resistance significantly limit their clinical application (Yap et al. 2021; Jardim et al. 2021; Marra et al. 2019). Overcoming these limitations to improve response rates and survival times in cancer patients is crucial. Identifying clinically feasible biomarkers to predict the efficacy of PD-1/PD-L1 antibodies is essential. We have identified three immunological studies suggesting that high expression of GZMK enhances patients' responsiveness to immunotherapy. Patients who responded effectively to immunotherapy showed higher GZMK expression levels than non-responders, with statistically significant differences (*P* < 0.05) (Fig. 7).

Numerous studies are currently exploring biomarkers for immunotherapy. Recent research has identified interferon-stimulated Ly6E^hi neutrophils as a blood marker for the efficacy of PD-1 antibody therapy in mice. These neutrophils trigger intratumoral activation of the STING (stimulator of interferon genes) signaling pathway and directly sensitize non-responsive tumors to PD-1 therapy, partly through IL12b-dependent activation of cytotoxic T cells (Lin et al. 2023). Applying these preclinical findings to a cohort of 109 patients with non-small cell lung cancer and melanoma, along with public data (*n* = 1440), demonstrated Ly6E^hi neutrophils' remarkable ability to accurately predict human immune therapy response, with an average AUC value exceeding 0.9 (Benguigui et al. 2024). Although current biomarker research primarily focuses on tumor PD-L1, microsatellite instability (MSI), and tumor mutational burden (TMB) (Lin et al. 2023), significant heterogeneity and controversies make clinical application challenging. The search for more efficient and clinically practical biomarkers continues to encounter significant obstacles.

Moreover, we found that GZMK increases sensitivity to tamoxifen (Figure S8D, E, F) while enhancing resistance to platinum-based drugs (Figure S8G, H, I). Tamoxifen and platinum-based drugs remain common in breast cancer treatment, offering new avenues for predicting drug efficacy. We conducted functional cell experiments after overexpressing GZMK in MDA-MB-231 cells (Fig. 8). These experiments showed that GZMK increased cell proliferation, promoted cell division, induced apoptosis, enhanced cell migration, and increased cell invasion (Fig. 8A, B, C, D, E). In vitro experiments have found that GZMK plays a complex role in MDA-MB-231 cells, which can not only increase cell proliferation and migration ability, but also increase apoptosis. The ultimate role in vivo still needs further animal model research. In the analysis of clinical data, we found that high expression of GZMK can improve the prognosis of patients (Figure S3–4), and it is speculated that the comprehensive role of GZMK in human body is to improve the prognosis of patients. Because the human body is a complex system, the role of GZMK in human body is diverse. In vitro experiments can only study part of its functions, and more rigorous experiments are needed to explore its comprehensive role. Conversely, a study in A375 and G361 cells (melanoma) found that GZMK overexpression inhibited cell proliferation and migration but did not affect apoptosis (Wu et al. 2021b). These findings further validate GZMK's role in breast cancer and provide a direction for subsequent mechanistic research.

Although we have found that GZMK plays an important role in breast cancer and has significant value in predicting breast cancer prognosis, it holds promise as a target for breast cancer immunotherapy or as a biomarker for predicting immunotherapy efficacy. However, our exploration through data from the TCGA database revealed that the sample size of breast cancer patients is still limited, requiring larger sample sizes to further validate our results. We also found a strong linear correlation between GZMK and PD-1 in breast cancer, providing a new therapeutic target for further exploration of breast cancer immunotherapy, especially in triple-negative breast cancer. Our research findings may provide other researchers with a novel research direction for breast cancer treatment and prognosis.

This article discusses the expression difference of GZMK in breast cancer, its impact on survival, the correlation analysis with immune markers, and the prediction of the efficacy of tumor immunotherapy. However, there is no in-depth study on the value of GZMK in breast cancer immunotherapy, especially through large sample clinical cohort study to verify the predictive value of GZMK for immunotherapy and clinical practical value. The classic IMpassion130 (Emens et al. 2021) and IMpassion131 (Gianni et al. 2022) randomized controlled phase III clinical trials were conducted to analyze the predictive value of PD-L1 for the efficacy of atezolizumab monoclonal antibody in advanced triple-negative breast cancer immunotherapy, and it was found that the immune combined with chemotherapy (OS = 25.4 months) significantly improved the prognosis of patients with PD-L1 positive patients compared with chemotherapy (OS = 17.9 months) (HR = 0.67, 95%CI: 0.53–0.86). In the KEYNOTE-522 study (Schmid et al. 2020), it was also found that pembrolizumab combined with chemotherapy significantly improved the pCR (Pathological Complete Response) rate of triple-negative breast cancer in PD-L1 positive patients (68.9%), while the pCR rate was 45.3% in negative patients. Next, we will further carry out clinical cohort studies on GZMK in neoadjuvant immunotherapy of breast cancer and immunotherapy of advanced breast cancer, stratify the efficacy of GZMK in high and low expression groups, and further analyze the accuracy of GZMK in breast cancer immunotherapy.

## Conclusions

In conclusion, our analysis of GZMK expression differences in breast cancer has provided initial insights into its potential mechanisms of action. High GZMK expression can improve OS and RFS in breast cancer patients, demonstrating its predictive value for patient survival and potential in predicting immunotherapy outcomes. The strong linear correlation between GZMK and PD-1 in breast cancer suggests a potential influence on T-cell differentiation and the T-cell receptor signaling pathway, offering a new therapeutic target for further exploration in breast cancer immunotherapy. Further laboratory-based research combined with clinical studies is necessary to validate these findings and to provide a more comprehensive understanding of GZMK's role in predicting prognosis and immunotherapy response in breast cancer patients.

### Supplementary Information

Below is the link to the electronic supplementary material.

Supplementary file1 (DOCX 3140 KB)

## Data Availability

No datasets were generated or analysed during the current study.
