# Supplementary material for: Exploring GZMK as a prognostic marker and predictor of immunotherapy response in breast cancer: unveiling novel insights into treatment outcomes
Source: J Cancer Res Clin Oncol. 2024 Jun 4;150(6):286. doi: 10.1007/s00432-024-05791-6 (PMC11150209; doi:10.1007/s00432-024-05791-6)
Supplement: Supplementary file 1 — Supplementary file1 (DOCX 3140 KB) [file 432_2024_5791_MOESM1_ESM.docx]

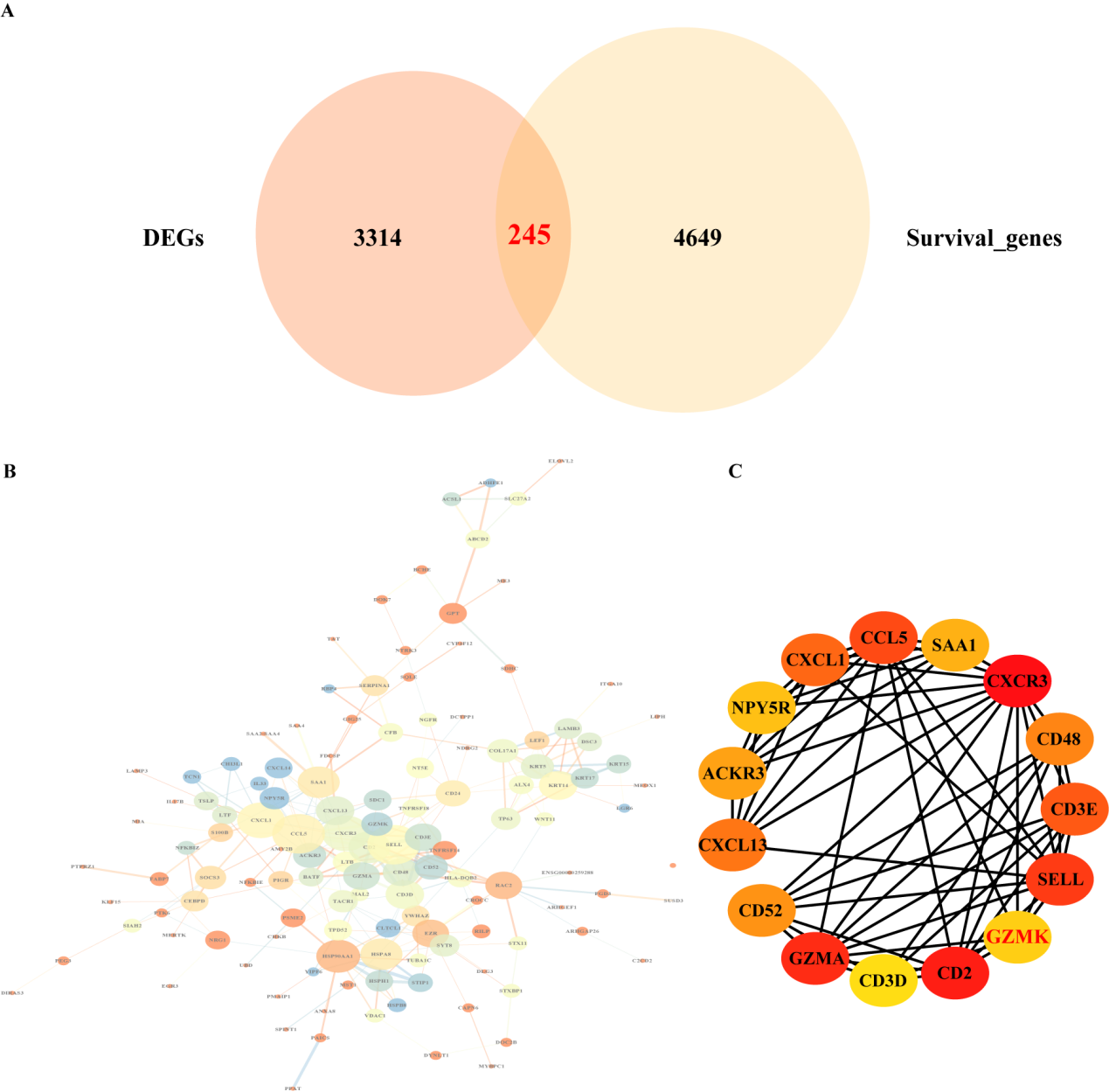


Figure S1 Presents the analysis results of differentially expressed genes (DEGs) and overall survival (OS)-related genes in breast cancer tissues compared to normal tissues from the TCGA database.

A. Venn diagram showing the overlap between differentially expressed genes and OS-related genes.

B. Protein-protein interaction (PPI) network diagram of the top 245 screened differential genes.

C. The top 15 protein molecules with the largest differential expression selected using Cytoscape software.


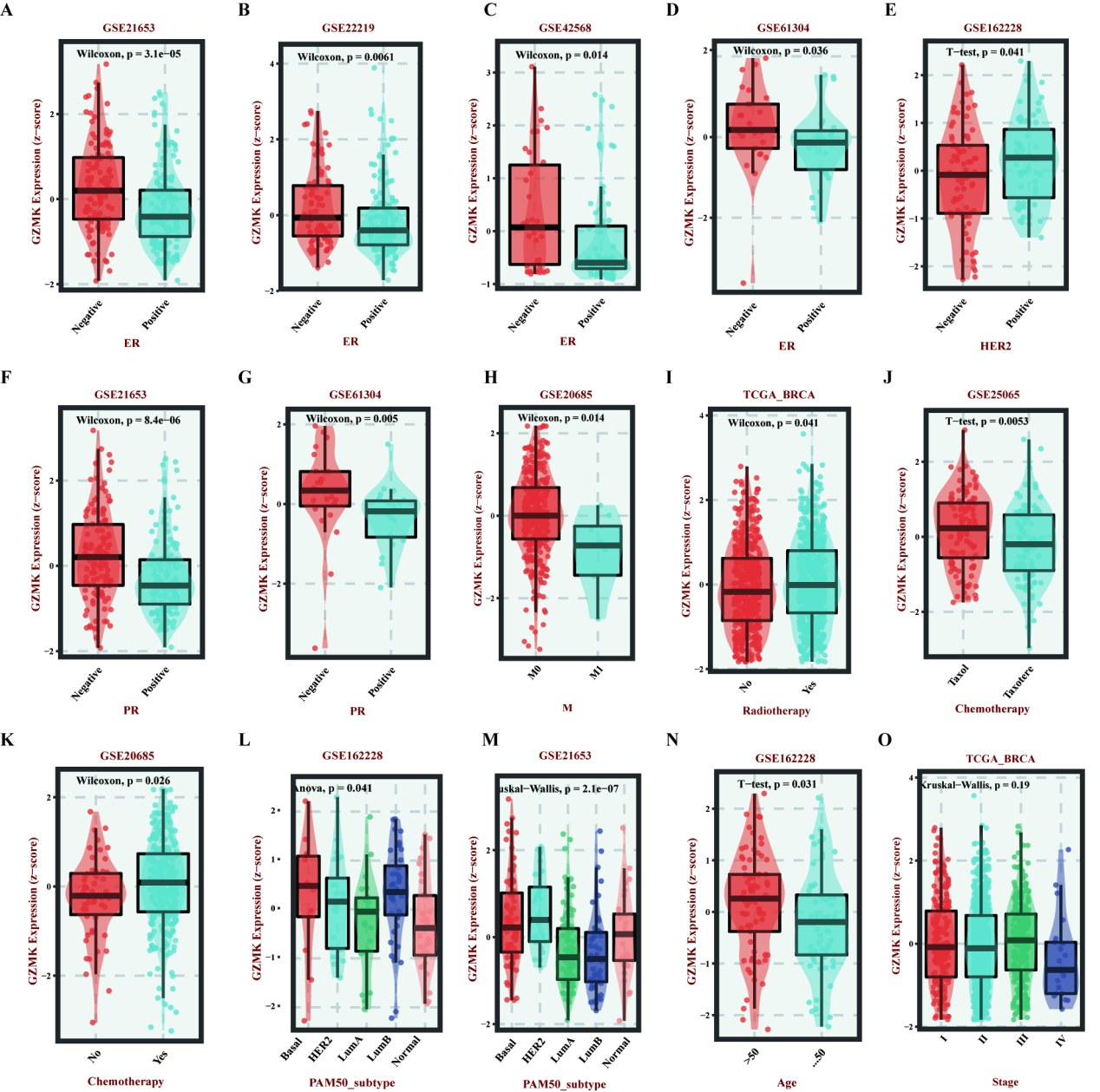


Figure S2 Presents the subgroup analysis results of GZMK in breast cancer.

A. Analysis results of ER (estrogen receptor) subtype from the GSE21653 dataset.

B. Analysis results of ER (estrogen receptor) subtype from the GSE22219 dataset.

C. Analysis results of ER (estrogen receptor) subtype from the GSE42568 dataset.

D. Analysis results of ER (estrogen receptor) subtype from the GSE61304 dataset.

E. Analysis results of HER2 (human epidermal growth factor receptor 2) subtype from the GSE16228 dataset.

F. Analysis results of PR (progesterone receptor) subtype from the GSE21653 dataset.

G. Analysis results of PR (progesterone receptor) subtype from the GSE61304 dataset.

H. Analysis results of patients with metastasis (M1) and without metastasis (M0) from the GSE20685 dataset.

I. Analysis results of GZMK expression levels in patients with effective and ineffective radiotherapy from the TCGA database.

J. Analysis results of GZMK expression levels in patients with effective and ineffective paclitaxel chemotherapy from the GSE25065 dataset.

K. Analysis results of GZMK expression levels in patients with effective and ineffective chemotherapy from the GSE20685 dataset.

L. Analysis results of GZMK expression levels in different molecular subtypes of breast cancer from the GSE162228 dataset.

M. Analysis results of GZMK expression levels in different molecular subtypes of breast cancer from the GSE21653 dataset.

N. Analysis results of GZMK expression levels in different age groups of breast cancer patients from the GSE162228 dataset.

O. Analysis results of GZMK expression levels in different stages of breast cancer from the TCGA database.


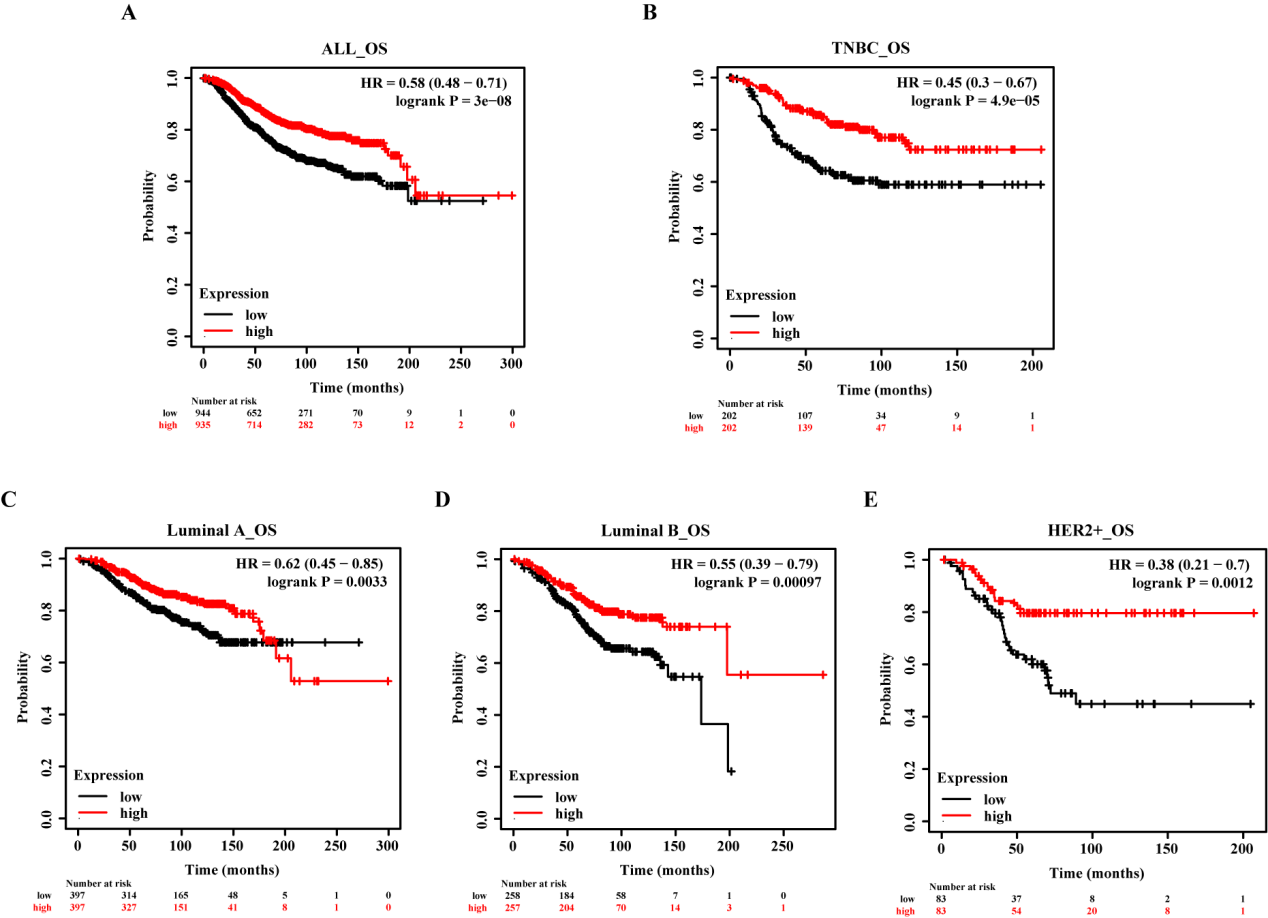


Figure S3 Illustrates the analysis of the relationship between GZMK and overall survival (OS) in breast cancer patients using the Kaplan-Meier Plotter database.

A. The impact of GZMK on OS in all breast cancer patients.

B. The impact of GZMK on OS in triple-negative breast cancer (TNBC) patients.

C. The impact of GZMK on OS in Luminal A subtype breast cancer patients.

D. The impact of GZMK on OS in Luminal B subtype breast cancer patients.

E. The impact of GZMK on OS in HER-2 positive breast cancer patients.


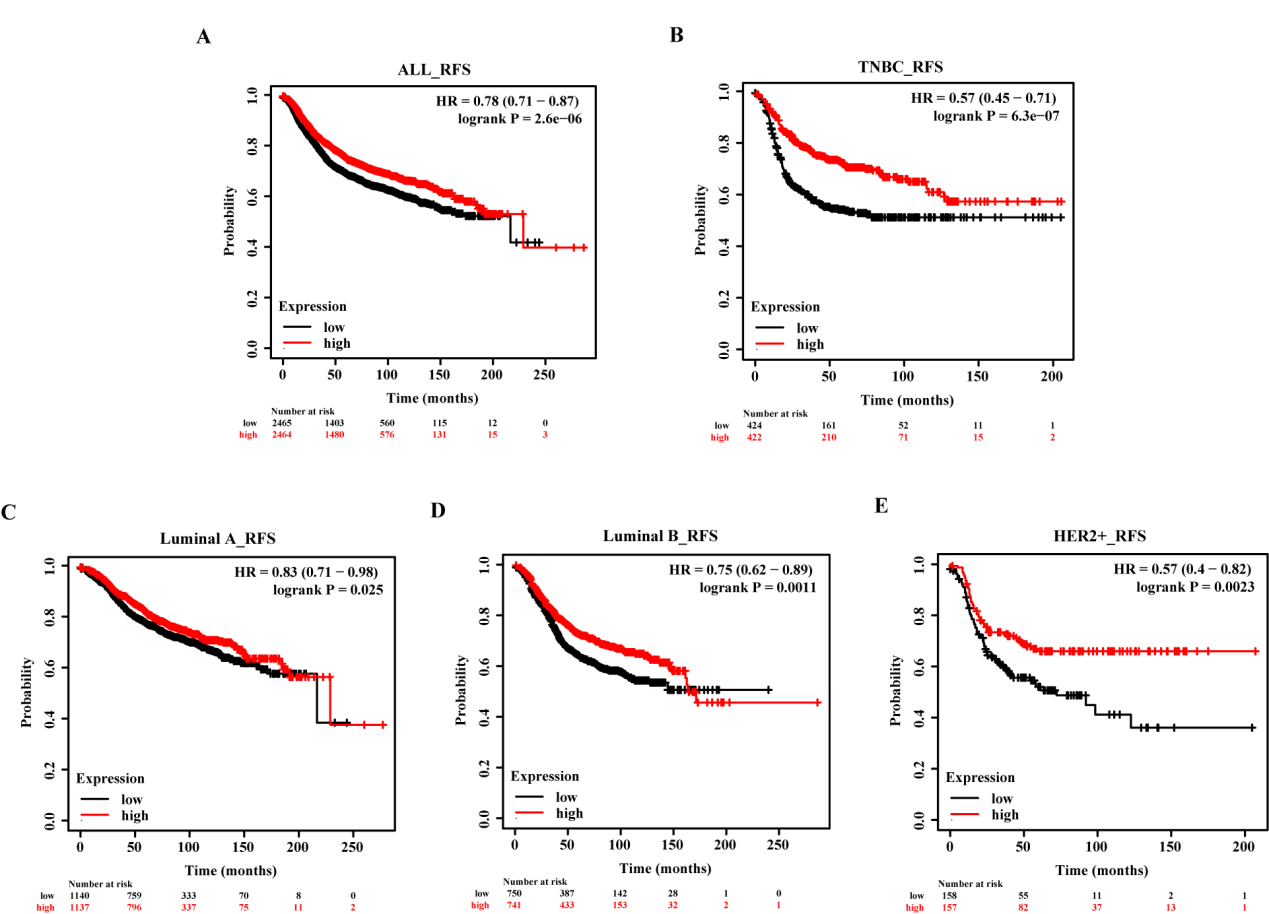


Figure S4 Depicts the analysis of the relationship between GZMK and relapse-free survival (RFS) in breast cancer patients using the Kaplan-Meier Plotter database.

A. The impact of GZMK on RFS in all breast cancer patients.

B. The impact of GZMK on RFS in triple-negative breast cancer (TNBC) patients.

C. The impact of GZMK on RFS in Luminal A subtype breast cancer patients.

D. The impact of GZMK on RFS in Luminal B subtype breast cancer patients.

E. The impact of GZMK on RFS in HER-2 positive breast cancer patients.


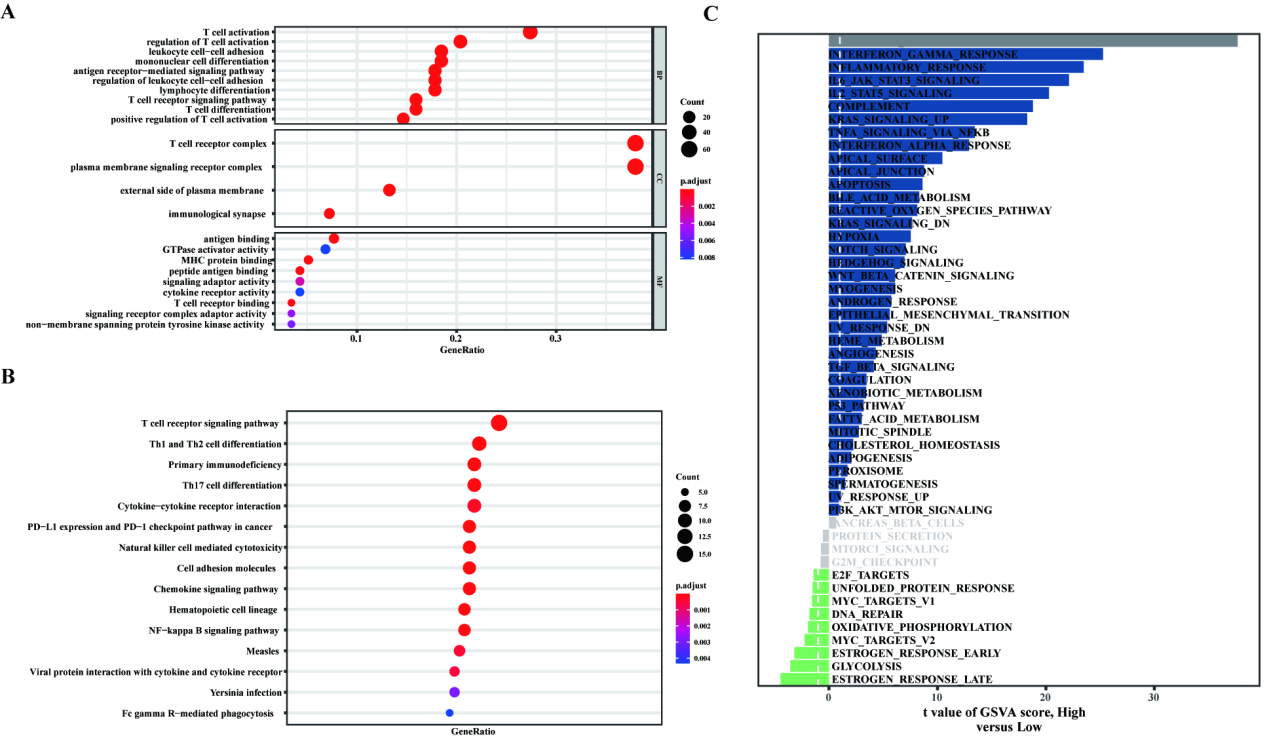


Figure S5 Presents the analysis of Gene Ontology (GO), Kyoto Encyclopedia of Genes and Genomes (KEGG), and Gene Set Variation Analysis (GSVA) for the intersection genes (245 genes) obtained from the differential expression genes and survival difference genes.

A. Results of the GO analysis.

B. Results of the KEGG analysis.

C. Results of the GSVA analysis.


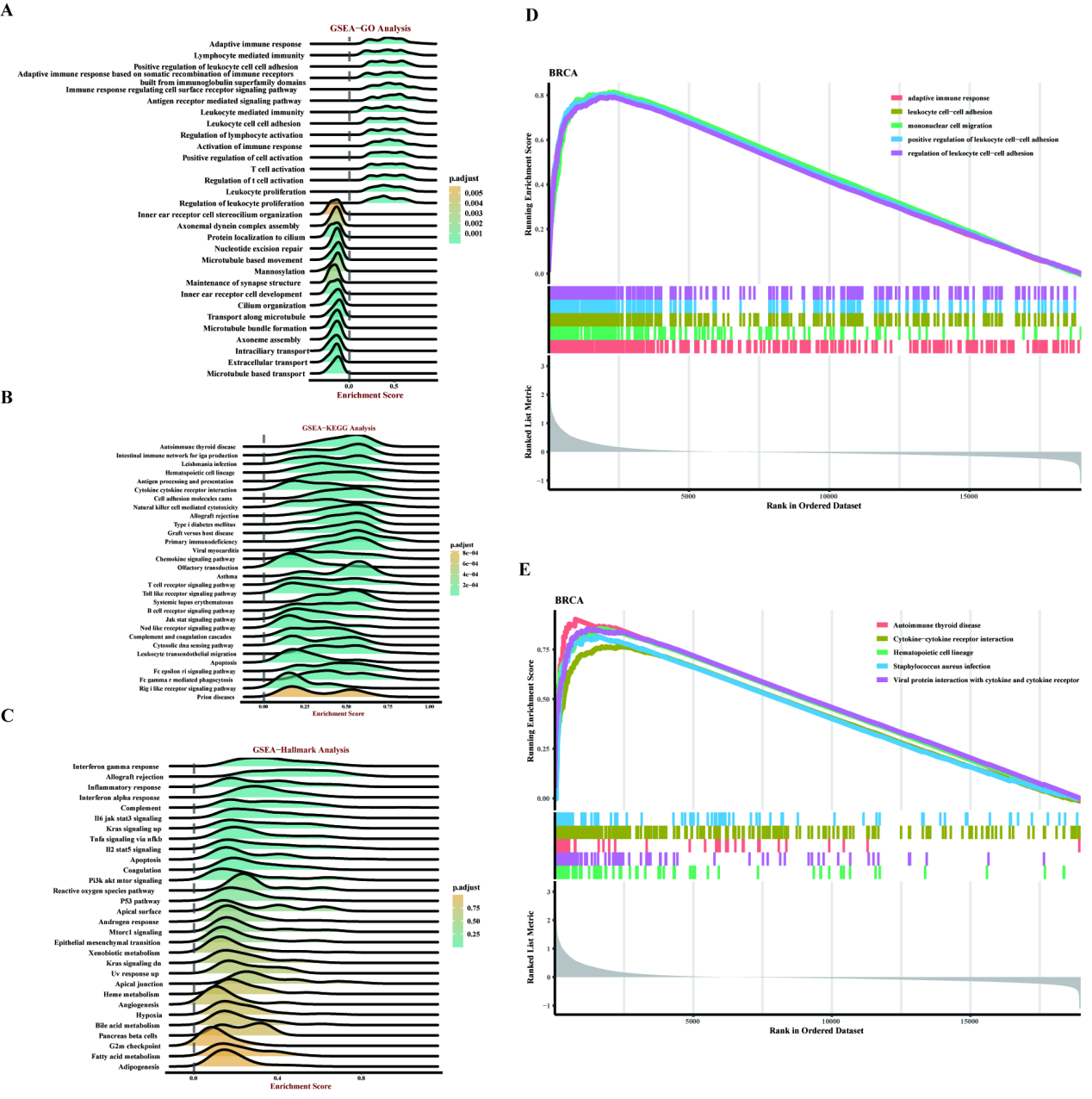


Figure S6 Presents the results of Gene Set Enrichment Analysis (GSEA) conducted on The Cancer Genome Atlas (TCGA) database and 19 Gene Expression Omnibus (GEO) datasets (GSE11121, GSE12093, GSE162228, GSE17705, GSE20685, GSE20711, GSE21653, GSE22219, GSE25055, GSE25065, GSE42568, GSE45255, GSE48390, GSE58812, GSE61304, GSE7390, GSE88770, GSE97342, and GSE9893).

A. GSEA-GO analysis ridge plot.

B. GSEA-KEGG analysis ridge plot.

C. GSEA-Hallmark analysis ridge plot.

D. TCGA database GSEA-GO analysis results.

E. TCGA database GSEA-KEGG analysis results.


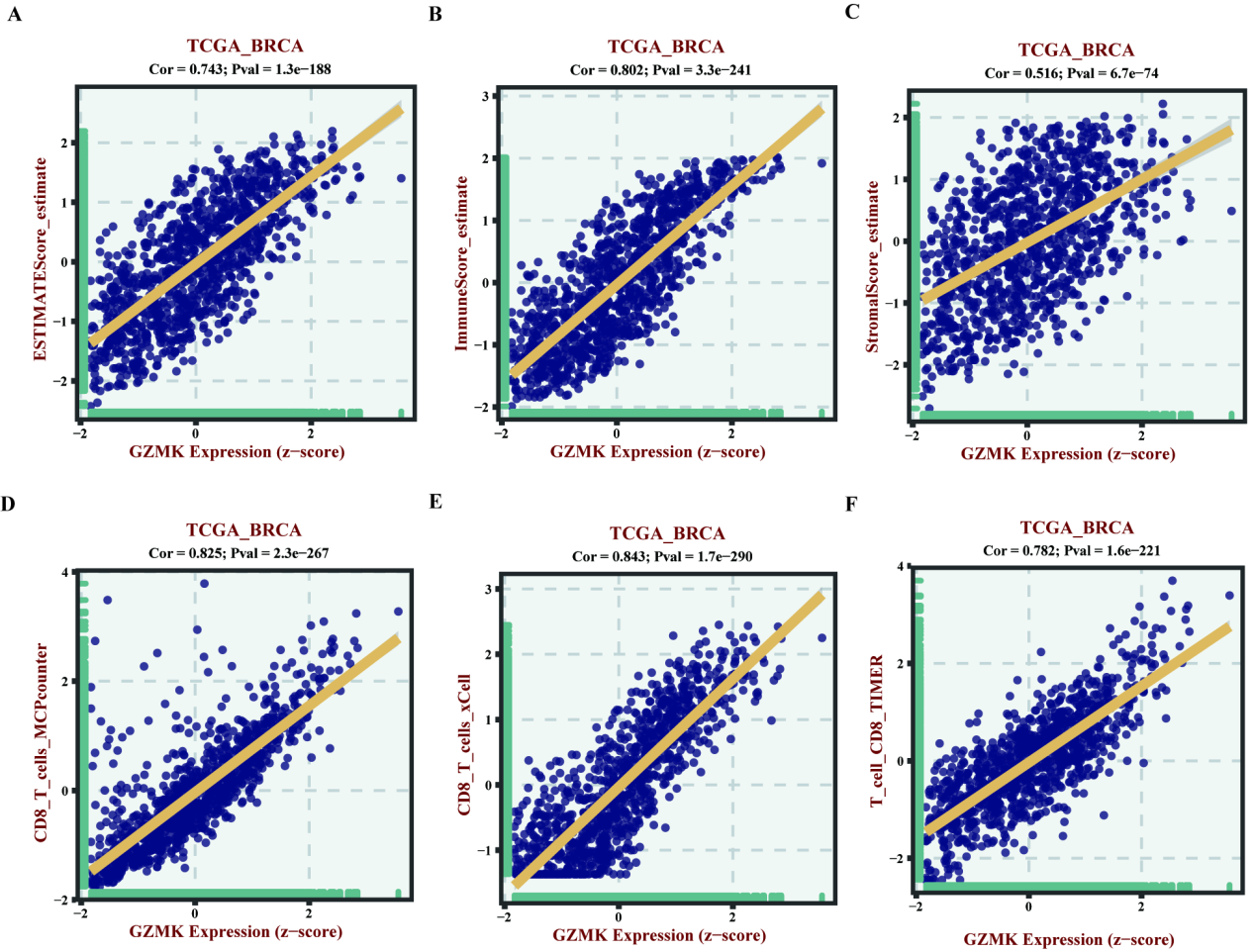


Figure S7 Shows the results of the analysis of the correlation between GZMK expression levels and major immune indicators in The Cancer Genome Atlas (TCGA) database.

A. Correlation between GZMK expression levels and ESTIMATE Score estimate.

B. Correlation between GZMK expression levels and Immune Score estimate.

C. Correlation between GZMK expression levels and Stromal Score estimate.

D. Analysis of the correlation between GZMK expression levels and CD8 T cells using the MCPcounter algorithm.

E. Analysis of the correlation between GZMK expression levels and CD8 T cells using the xCell algorithm.

F. Analysis of the correlation between GZMK expression levels and CD8 T cells using the TIMER algorithm.


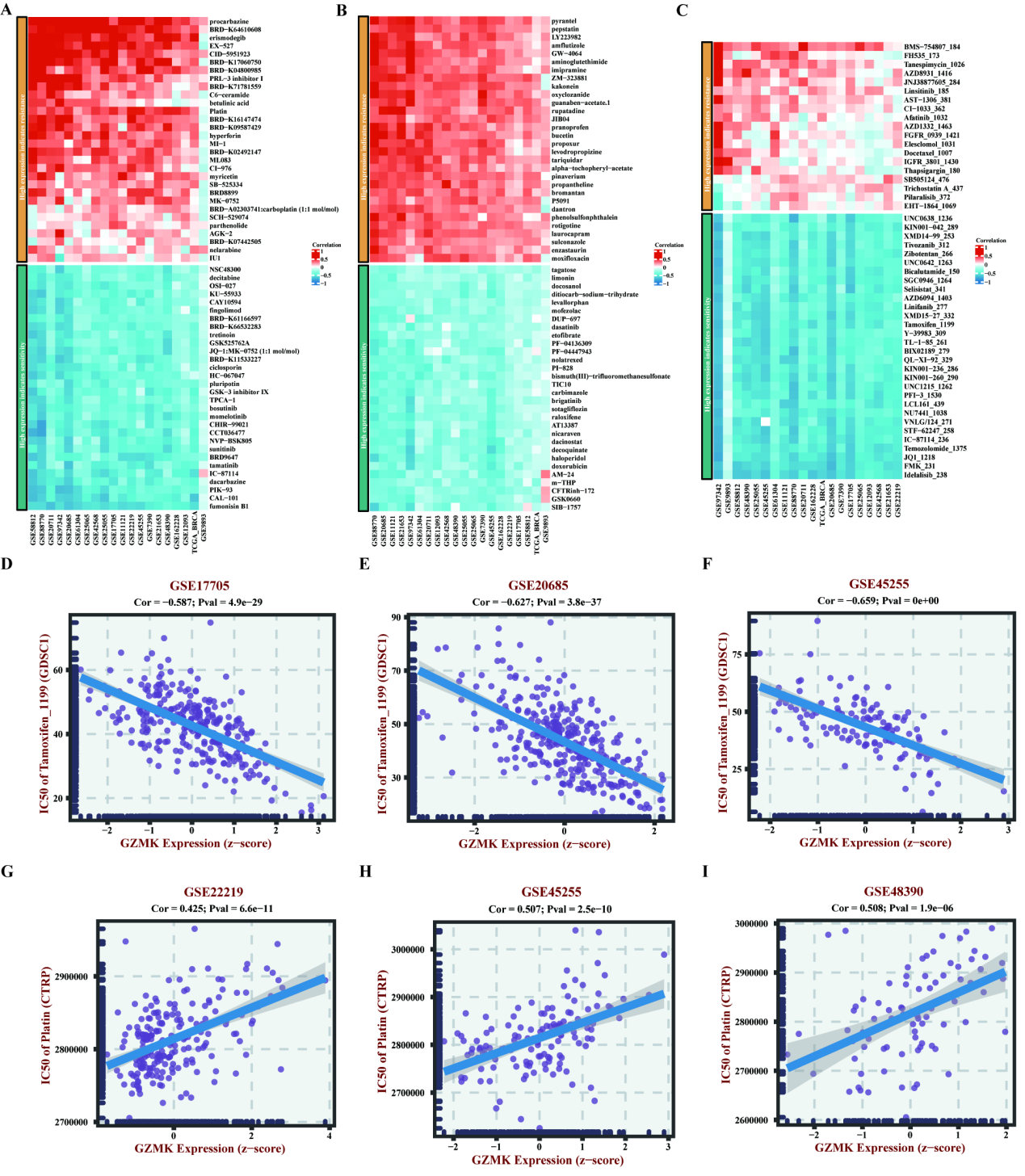


Figure S8 Presents the results of the drug sensitivity analysis of GZMK.

A. Analysis results from the GDSC_v1 database.

B. Analysis results from the CTRP database.

C. Analysis results from the PRISM database.

D. Analysis results of the correlation between GZMK expression levels and tamoxifen IC50 in the GSE17705 dataset.

E. Analysis results of the correlation between GZMK expression levels and tamoxifen IC50 in the GSE20685 dataset.

F. Analysis results of the correlation between GZMK expression levels and tamoxifen IC50 in the GSE45255 dataset.

G. Analysis results of the correlation between GZMK expression levels and platinum IC50 in the GSE22219 dataset.

H. Analysis results of the correlation between GZMK expression levels and platinum IC50 in the GSE45255 dataset.

I. Analysis results of the correlation between GZMK expression levels and platinum IC50 in the GSE48390 dataset.

Table S1 Clinicopathological characteristics of breast cancer patients divided into High- and Low-GZMK expression groups from TCGA

| **Characteristic** | **GZMK_Low, N = 265** | **GZMK_High, N = 265** | ***P*-value2** |
| --- | --- | --- | --- |
| Patient age |  |  | 0.602 |
| <58 | 132 (50%) | 139 (52%) |  |
| >=58 | 133 (50%) | 126 (48%) |  |
| Pathologic_T |  |  | 0.050 |
| T1 | 65 (25%) | 67 (25%) |  |
| T2 | 172 (65%) | 160 (60%) |  |
| T3 | 16 (6.0%) | 32 (12%) |  |
| T4 | 12 (4.5%) | 6 (2.3%) |  |
| Pathologic_N |  |  | 0.128 |
| N0 | 121 (46%) | 101 (38%) |  |
| N1 | 106 (40%) | 112 (42%) |  |
| N2 | 38 (14%) | 52 (20%) |  |
| Pathologic_M |  |  | 0.724 |
| M0 | 260 (98%) | 262 (99%) |  |
| M1 | 5 (1.9%) | 3 (1.1%) |  |
| Gender |  |  | >0.999 |
| female | 260 (98%) | 261 (98%) |  |
| male | 5 (1.9%) | 4 (1.5%) |  |
| Race |  |  | 0.269 |
| asian | 19 (7.2%) | 26 (9.8%) |  |
| black or african american | 34 (13%) | 34 (13%) |  |
| not reported | 37 (14%) | 24 (9.1%) |  |
| white | 175 (66%) | 181 (68%) |  |
| Stage |  |  | 0.510 |
| stage Ⅰ | 39 (15%) | 34 (13%) |  |
| stage Ⅱ | 167 (63%) | 161 (61%) |  |
| stage Ⅲ | 54 (20%) | 67 (25%) |  |
| stage Ⅳ | 5 (1.9%) | 3 (1.1%) |  |
| Subtype |  |  | 0.018* |
| Basal | 43 (16%) | 56 (21%) |  |
| Her2 | 13 (4.9%) | 25 (9.4%) |  |
| LumA | 134 (51%) | 133 (50%) |  |
| LumB | 75 (28%) | 51 (19%) |  |
| PD-1 |  |  | <0.001** |
| <median | 218 (82%) | 47 (18%) |  |
| >=median | 47 (18%) | 218 (82%) |  |
| PD-L1 |  |  | <0.001** |
| <median | 190 (72%) | 75 (28%) |  |
| >=median | 75 (28%) | 190 (72%) |  |
| CTLA-4 |  |  | <0.001** |
| <median | 202 (76%) | 63 (24%) |  |
| >=median | 63 (24%) | 202 (76%) |  |

Note: For continuous variables, comparison between groups was performed using t test, and for categorical variables, comparison between groups was performed using Fisher's exact probability test.**P*<0.05。*P<0.05，**P<0.001。

Table S2 Clinicopathologic characteristics of patients with breast cancer

| Characteristic | N = 131 |
| --- | --- |
| Age |  |
| <54 | 72 (55%) |
| >=54 | 59 (45%) |
| Location |  |
| left | 60 (46%) |
| right | 71 (54%) |
| Differentiation |  |
| high | 9 (6.9%) |
| low | 29 (22%) |
| mediate | 93 (71%) |
| Tumor |  |
| T1 | 57 (44%) |
| T2 | 65 (50%) |
| T3 | 9 (6.9%) |
| Positive lymph nodes |  |
| N0 | 70 (53%) |
| N1 | 42 (32%) |
| N2 | 13 (9.9%) |
| N3 | 6 (4.6%) |
| Pathological staging |  |
| ⅠA | 36 (27%) |
| ⅡA | 43 (33%) |
| ⅡB | 32 (24%) |
| ⅢA | 13 (9.9%) |
| ⅢC | 7 (5.3%) |
| ER |  |
| negative | 25 (19%) |
| positive | 106 (81%) |
| PR |  |
| negative | 38 (29%) |
| positive | 93 (71%) |
| HER2 |  |
| 0 | 43 (33%) |
| 1+ | 43 (33%) |
| 2+ | 29 (22%) |
| 3+ | 16 (12%) |
| Ki-67 |  |
| <20 | 65 (50%) |
| >=20 | 66 (50%) |
| Molecular Typing |  |
| HER-2 positive | 6 (4.6%) |
| LuminalA | 34 (26%) |
| LuminalB | 73 (56%) |
| TNBC | 18 (14%) |
| AR |  |
| negative | 20 (15%) |
| positive | 111 (85%) |
| Blood Vessel |  |
| negative | 45 (34%) |
| positive | 86 (66%) |
| GZMK |  |
| Low | 80 (61%) |
| High | 51 (39%) |

Table S3 Clinicopathologic characteristics of patients with breast cancer from TCGA

| Characteristic | N = 530 |
| --- | --- |
| Age |  |
| <58 | 271 (51%) |
| >=58 | 259 (49%) |
| Pathologic_T |  |
| T1 | 132 (25%) |
| T2 | 332 (63%) |
| T3 | 48 (9.1%) |
| T4 | 18 (3.4%) |
| Pathologic_N |  |
| N0 | 222 (42%) |
| N1 | 218 (41%) |
| N2 | 90 (17%) |
| Pathologic_M |  |
| M0 | 522 (98%) |
| M1 | 8 (1.5%) |
| Gender |  |
| female | 521 (98%) |
| male | 9 (1.7%) |
| Race |  |
| asian | 45 (8.5%) |
| black or african american | 68 (13%) |
| not reported | 61 (12%) |
| white | 356 (67%) |
| Stage |  |
| stage Ⅰ | 73 (14%) |
| stage Ⅱ | 328 (62%) |
| stage Ⅲ | 121 (23%) |
| stage Ⅳ | 8 (1.5%) |
| Subtype |  |
| Basal | 99 (19%) |
| Her2 | 38 (7.2%) |
| LumA | 267 (50%) |
| LumB | 126 (24%) |
| GZMK |  |
| Low | 265 (50%) |
| High | 265 (50%) |
